# Modifications of microbiome-derived cell-free RNA in plasma discriminates colorectal cancer samples

**DOI:** 10.1038/s41587-025-02731-8

**Published:** 2025-07-08

**Authors:** Cheng-Wei Ju, Ruitu Lyu, Han Li, Jiangbo Wei, Alberto J Parra Vitela, Urszula Dougherty, Akushika Kwesi, Alexander Luna, Xuanhao Zhu, Shenghai Shen, Yunzheng Liu, Liangliang Wang, Xiaolong Cui, Yuzhi Xu, Bochen Jiang, Yiyi Ji, Peng Xia, Diana C. West-Szymanski, Chenxi Sun, Yuhao Zhong, Chang Ye, Angelica Moran, Christopher Lehmann, Eric Pamer, Wei Zhang, Marc Bissonnette, Li-Sheng Zhang, Chuan He

**Affiliations:** 1 Department of Chemistry, The University of Chicago, Chicago, IL, USA; 2 Howard Hughes Medical Institute, The University of Chicago, Chicago, IL, USA; 3 Pritzker School of Molecular Engineering, The University of Chicago, Chicago, IL, USA; 4 Colon and Rectal Surgery, Advocate Lutheran General Hospital, Park Ridge, IL, USA; 5 Department of Medicine, The University of Chicago, Chicago, IL, USA; 6 Division of Life Science, The Hong Kong University of Science and Technology, Kowloon, Hong Kong SAR, China; 7 Department of Chemistry, The Hong Kong University of Science and Technology, Kowloon, Hong Kong SAR, China; 8 Division of Biology and Biological Engineering, California Institute of Technology, Pasadena, CA, USA; 9 The Laboratory of Microbiome and Microecological Technology, Institute of Microbiology, Chinese Academy of Sciences, Beijing, China; 10 Department of Chemistry, New York University, New York, NY, USA; 11 Department of Preventive Medicine and The Robert H. Lurie Comprehensive Cancer Center, Northwestern University Feinberg School of Medicine, Chicago, IL, USA

## Abstract

Circulating cell-free RNA (cfRNA) in plasma represents a promising avenue for cancer detection. We report Low-Input Multiple Methylation Sequencing (LIME-seq), a method for profiling modification patterns in cfRNA, enabling the detection of diverse tRNAs and small non-coding RNAs derived from both the human genome and the microbiome. RNA modification patterns in microbiome-derived cfRNA accurately reflect host microbiota activity and hold potential for the early detection of colorectal cancer.

In precision medicine, circulating cell-free DNA (cfDNA) has emerged as an increasingly important analyte, providing valuable clinical information and non-invasive diagnoses across various domains^1–3^. A key strategy for cancer diagnosis involves identifying the changes in tumor-derived cell-free DNA (cfDNA)^4,5^. However, cfDNA concentrations in plasma can be exceedingly low in early-stage cancer patients, resulting in reduced sensitivity and specificity^6^. While epigenetics-based cfDNA sequencing has shown promise^7,8^, the low concentration of cfDNA at the early cancer stages remains a significant challenge^9^. In addition, the epigenetic alterations found in cancer may also be present in non-cancer tissues, adding another layer of complexity to the diagnostic process^10^.

Cell-free RNA (cfRNA) released from apoptotic cells provides an alternative analyte for non-invasive diagnosis and detection of various diseases^11–13^. Changes in cellular RNA transcription are dynamic processes that can serve as indicators of disease pathobiology^12–15^. While overexpression of tumor-specific transcripts may enhance tumor-derived RNA signals, the limited cell death in the early stages of cancer is again a major challenge. Cell-free mRNAs are susceptible to degradation if not protected by protein binding and are present in low quantities in clinically feasible samples (*e.g*., a few mL of plasma), complicating their amplification and detection^16^. Additionally, contamination from unrelated cells can markedly alter the abundance of cell-free mRNAs, further complicating detection specificity.

The presence of site-specific RNA modifications and changes in their stoichiometry add new layers of information beyond RNA sequences. With the development of new methods for sequencing RNA modifications^17–22^, we hypothesized that the changes in RNA modifications from tumor- or tumor microenvironment (TME)-derived transcripts could be distinct from those in non-tumor cells. We therefore investigated cfRNA modifications in clinical samples. We observed that a stable amount of total RNA (~5–10 ng) could be isolated from 1 mL of plasma. Short RNA fragments (~50 nt) are the predominant forms in the isolated cfRNAs, with sizes similar to those of transfer RNAs (tRNAs) (Fig. 1a). tRNAs are highly structured and more resistant to nuclease-mediated degradation than other RNA species. Based on the observed RNA size profiles, other structured non-coding RNAs (ncRNAs) or tRNA fragments (tRF) may also be present (Fig. 1a). These RNAs could be bound by tRNA synthetases or RNA-binding proteins (RBPs), providing additional protection against degradation in plasma. Given that tRNAs are known to be heavily modified, we speculated that changes in tRNA modification stoichiometry might offer additional clues for human disease diagnosis (*e.g*. detection of early-stage cancer).

The numerous internal modifications (frequent in tRNAs) may obstruct the read-through of reverse transcriptase; however, these modification (most are methylations) patterns in cellular tRNAs carry valuable information^23,24^. We previously reported a method to sequence these methylations that block Watson-Crick base pairing^21^. We subsequently optimized the protocol by removing the demethylation step, which previously caused RNA degradation, and developed Low-Input Multiple Methylation Sequencing (LIME-seq). This method enables simultaneous detection of site-specific RNA modifications (e.g., m^1^A, m^3^C, m^1^G, m^2^_2_G, m^3^U, inosine, etc.) across multiple RNA species in ultralow-input cfRNAs, while also monitoring stoichiometry changes of these modifications (Fig. 1b)^21^. LIME-seq maps and quantifies these modifications via base mutation (base misincorporation) generated during reverse transcription (RT), using human immunodeficiency virus reverse transcriptase (HIV-RT). LIME-seq employs cDNA 3’-ligation after RT and utilizes HIV-RT to ensure read-through of RNA methylations while inducing mutation signatures for quantification^25^. These features allow us to measure the abundance of individual small RNAs with greater precision, and to quantitatively reveal the landscape of major RNA methylations on cfRNAs. The RNA/cDNA ligation strategy in LIME-seq also ensures the capture of all short RNA species in plasma, which are often lost in typical RNA-seq libraries that use commercial kits. To validate LIME-seq, we applied it to small RNAs (<200 nt) isolated from HepG2 cells. For cytoplasmic tRNAs, LIME-seq detected two tRNAs with m^1^A_9_, forty-four with m^1^A_58_, seven with m^3^C_32_, ten with m^1^G_9_, ten with m^1^G_37,_ and twenty-one with m^2^_2_G_26_, along with modification stoichiometry information (Supplementary Data Fig. 1a–f)^21,26,27^. Intact read coverage profiles were observed at tRNA regions through LIME-seq, demonstrating its capability to measure tRNA abundance as well (Extended Data Fig. 1a). We also performed LIME-seq using 1.5–50 ng of HepG2 small RNA and confirmed high consistency between technical replicates (*r* = 0.990) and minimal effect from RNA input variation (*r* > 0.96 between 50 ng and 1.5 ng, Extended Data Fig. 1b–f).

We next applied LIME-seq to cfRNAs isolated from 36 non-cancer human plasma samples. Notably, 19 out of the top 50 highly expressed transcripts were cytoplasmic tRNAs, which accounted for ~13% of the total reads mapped to the human genome (GRCh38). This finding supports the notion that tRNAs are major components of cfRNA in human plasma (Fig. 1c–d). LIME-seq also captured abundant RNA fragments from rRNAs, *Alu* elements, LINEs, snRNAs, mRNAs, and lncRNAs. We observed intact read coverage profiles in most annotated cytoplasmic tRNA regions (including mitochondrial tRNA regions, Fig. 1g), indicating the preservation of cell-free tRNAs in human plasma. In contrast, only fragments were detected for other abundant cfRNA species, such as snRNAs, Y RNAs, 7SK RNAs, and rRNAs (Fig. 1e–f and Extended Data Fig. 2a–e). The presence of intact tRNAs aligns with their highly structured nature or potential protection by tRNA synthases. Furthermore, we validated that LIME-seq comprehensively captured human tRNA methylation sites in cfRNAs, providing methylation stoichiometry information for both cytoplasmic and mitochondrial tRNAs as well as fragments from rRNAs (Extended Data Fig. 2f and Supplementary Data Fig. 2a–g).

Notably, while we observed that ~60-80% mapped cfRNA reads aligned with the human genome, we also found that a significant fraction (~20–40%) aligned with the microbial genomes (Fig. 1h). These microbiome-derived sequences, which ranges from ~30–50 nt in length, allowed us to study their bacterial origins. Over 60% of these reads could be mapped to five major bacterial orders, we also detected several hundred distinct gut microbes based on rRNA fragments and tRNA sequences, with prominent representation from the orders *Bacillales, Xanthomonadales, Burkholderiales, Enterobacterales, Pseudomonadales* (Fig. 1i and Extended Data Fig. 3a). Due to the short read lengths and high sequence homology among species, resolution below the Order level may not be accurate (Extended Data Fig. 3b–e). To further validate that LIME-seq can accurately identify microbiome RNAs in plasma, we isolated small RNAs from lab-cultured *S. maltophilia,* which is a model organism for the order Xanthomonadales, and conducted LIME-seq. We confirmed the consistency between plasma cfRNA and the RNA from lab-cultured microbes in terms of both read coverage and methylation patterns; skin samples served as negative controls, showing poor read coverage or distinct read patterns when aligned to the *S. maltophilia* genome (Extended Data Fig. 3e–h). We also applied the AlkB-mediated demethylation of small RNAs isolated from cultured microbes and confirmed that the methylation sites detected in these RNAs were also present in cfRNA from plasma. To assess potential skin microbe contamination, we collected multiple tubes of blood samples from the same patient without changing the needle between blood draws. The microbial profiles remained consistent across samples, suggesting minimal skin microbe contamination which would be expected to enrich in the first tube.

Since we are particularly intrigued by the potential to monitor commensal microbial species through microbiome-derived cfRNAs; the abundance and methylation patterns revealed by LIME-seq may reflect the dynamic status of host microbiomes. Given the increasing evidence of gut microbiome’s role in colorectal cancer (CRC) initiation and progression, we asked whether cfRNA profiled by LIME-seq could be a non-invasive detection method for CRC patients^28^.

We collected plasma samples from 27 CRC patients (5 at stage 0, 6 at stage I, 8 at stage II, 8 at stage III) and 36 age- and sex-matched non-cancer individuals as controls. LIME-seq was performed using cfRNAs isolated from ~600 uL of plasma from each participant (Supplementary Table 1). Overall, we observed that ~30% of mapped reads aligned with the microbial genome for both CRC patients and non-cancer controls (Supplementary Data Fig. 3a), and the most abundant microbial species were common to both groups (Supplementary Data Fig. 3b–d). Although differential host cfRNA profiling revealed a difference in RNA levels of several host RNA species between CRC patients and controls (Fig. 1j and Extended Data Fig. 4a–b), the expression levels and mutation signatures of these host snRNAs, tRNAs, and rRNAs were not able to significantly classify CRC patients from controls (Extended Data Fig. 4c–i). We next collected tumor and adjacent normal tissues from four patients and performed LIME-seq on isolated small RNAs. We indeed observed tRNA modification differences in tumor versus normal tissue samples. However, these tRNA methylation signatures in plasma cfRNA were not able to distinguish CRC patients from non-cancer controls (Extended Data Fig. 4j–n). We suspect cell-free host tRNAs may be derived from a range of different cell types, which potentially displayed different tRNA methylation levels based on cell type and cell status and masked the signatures of those from tumor tissues. In contrast, the well-developed Kraken2 methodology enabled us to estimate the relative abundance of various microbial species using the mapped reads from LIME-seq. Although species-level resolution may be limited, the analysis revealed distinct abundances patterns between CRC patients and non-cancer controls, suggesting their potential as biomarkers for CRC diagnosis (Fig. 1j)^29^.

Previous studies have shown that cfDNA and cfRNA from microorganisms in plasma may reflect the abundance of various microbes, suggesting their potential as noninvasive biomarkers for cancer detection^30–32^. Therefore, we established a statistical model based on these microbiome-related reads sequences (Extended Data Fig. 5a). The detection performance of this model was evaluated using the leave-one-out cross-validation (LOOCV), given the relatively small sample size. An area under the curve (AUC) of 0.77 was achieved (Extended Data Fig. 5b), showing 56% sensitivity and 97% specificity at the cutoff that maximized the Youden’s index. This finding corroborates results from a previous report that also utilized plasma cell-free RNAs derived from human and microbes (Extended Data Fig. 5c)^31^, validating our approach despite the limited sample size. Larger cohorts of clinical samples are warranted in the future for further improvement.

We next investigated whether changes in the distribution of methylation site distribution or methylation stoichiometry in fragmented cfRNA could enhance discrimination power beyond cfRNA abundance changes. Using *S. maltophilia* as an example, LIME-seq clearly detected methylation sites such as m^1^G_37_ in tRNA-Pro-TGG, tRNA-Pro-CGG and tRNA-Leu-CAG in plasma cfRNA and validated these sites by AlkB demethylation treatment in lab-cultured microbes (Fig. 2a and Extended Data Fig. 3e–h). Our analysis revealed differential methylation patterns, including both up- and down-alteration of methylation levels, across various methylated sites in cell-free tRNAs and other small RNA fragments when comparing colorectal cancer (CRC) patients to non-cancer individuals. Notably, the majority of methylation sites exhibited increased modification levels in the CRC samples (Extended Data Fig. 6a). Higher modification levels in microbiome RNA may correlate with increased metabolic activity and external stress, suggesting dysregulation of microbiomes in CRC patients^33–37^. Further, a heatmap plot confirmed the presence of differential methylation sites in microbiome-derived cfRNAs between CRC patients and non-cancer individuals (Fig. 2b). We were able to distinguish the mutational signatures from RNA modifications and single nucleotide polymorphisms (SNPs) by identifying mixed mutational signatures that were only present at RNA modification sites during RT (Extended Data Fig. 6b). RNA modifications constitute a significant component and feature a notably higher ratio of sites with statistically meaningful differences (*P* < 0.05, Extended Data Fig. 6c). These results strongly suggest the potential of microbiome-derived cfRNA methylation patterns as biomarkers for CRC detection. Principal Component Analysis (PCA) revealed that high-dimensional data are crucial for distinguishing mutation signatures between CRC patients and non-cancer individuals (Extended Data Fig. 6d–e). This insight led us to adopt a support vector machine (SVM) for our diagnostic model, which effectively separates patient and non-cancer classes using a hyperplane (Extended Data Fig. 6f)^38,39^. By applying LIME-seq to cfRNA samples, we were able to analyze mutation patterns of the microbiome-derived cfRNA, thereby generating predictive indicators for CRC (Extended Data Fig. 6g).

Our statistical model, validated using LOOCV, demonstrated exceptional predictive ability for classifying CRC patients, achieving an AUC of 0.98 and an accuracy rate of 0.95 (Fig. 2c and Extended Data Fig. 7a). This represents a significant improvement compared to the model based solely on microbial abundance, which attained an AUC of only 0.77 (Fig. 2c). Furthermore, our statistical model enabled the application of these mutation signatures in distinguishing CRC stages (Fig. 2d). where remarkably high accuracy was observed even for early stages. This sensitivity may be attributed to the microbiota’s responsiveness to abnormal cells, even at stage 0^40–42^. Recognizing that LOOCV may overestimate our model’s performance in real-world scenarios, we employed more rigorous validation methods, including bootstrapping and k-fold cross-validation, both utilizing a 25% validation set (Extended Data Fig. 7b). Under these stringent validation frameworks, our mutation-signature-based model maintained high accuracy with an AUC of 0.92 (Confidence Intervals (CI) 95%: 0.82-1.00, Extended Data Fig. 7c–e). At the optimal cutoff determined by maximizing Youden’s index, our model exhibited a sensitivity of 0.93 (CI 95%: 0.89-0.97), a specificity of 0.92 (CI 95%: 0.88-0.97), with an accuracy of 0.92 (CI 95%: 0.89-0.95). This performance significantly outperforms the model that relied solely on microbial species abundances as biomarkers, which attained an AUC of 0.68 (CI 95%: 0.43-0.86, Extended Data Fig. 7c–e). In comparison to the FDA-approved blood-based CRC test (SEPT9 promoter methylation), which had ~70% sensitivity in CRC detection, our results suggest a substantial potential for significantly enhancing detection accuracy for early CRC^43–45^.

We lastly accessed the important methylation sites identified in our model using Shapley Additive explanations (SHAP), a cooperative game theory-based approach for interpreting statistical models (Fig. 2e–g). Compared to most sequences detected in total cfRNA, we found that tRNA sequences and regions near modification sites could provide sufficient resolution to classify microbes at the family to even genus level, likely due to their lower sequence conservation. Among the top 10 important microbes, *Burkholderia* (*Burkholderiaceae*) emerged as a key target with upregulated methylation level and is distinctly enriched in CRC patients^46^; some members of *Microbacterium (Microbacteriaceae)*, also as top targets, have been implicated in infections in immunocompromised individuals, such as cancer patients, and has been found to display increased methylation level in CRC tumor tissues^47–49^. Conversely, *Clostridium (Clostridiaceae)*, which exhibited reduced methylation level in CRC patients, have been studied as a target for anticancer treatment^50–52^. Notably, 18 out of the 26 most significant genera identified by our model corresponded with those found in previous research (Supplementary Table 2–3). Additionally, we successfully traced changes of the microbiome across different stages of CRC (Fig. 2g and Extended Data Fig. 7f–h). We further focused on microbes known to be enriched in CRC patients by aligning reads to speicifc microbes, including *Peptostreptococcus anaerobius*, *Prevotella intermedia*, and *Parvimonas micra*^53–56^. We observed high mutation levels among differentially methylated sites in cfRNA from CRC patients, suggesting a connection between RNA methylation levels and elevated microbiome activity (Extended Data Fig. 7i).

To evaluate the robustness of these biomarkers under clinical conditions, we collected plasma from two healthy individuals and stored them at 4°C for 2, 8, and 24 hours before plasma extraction (Extended Data Fig. 8a). We observed a high correlation of mutational signatures in microbiome reads maintained after 8 hours of storage (r > 0.9, Extended Data Fig. 8b). Although an acceptable correlation remained after 24 hours (r > 0.85), there was a significant increase in the human genome fraction, likely due to the leakage of blood cells over time (Extended Data Fig. 8c), leading to large variations of host cfRNA reads (Extended Data Fig. 8d).

From an initial set of 330 features, we refined the model to 12 highly expressed sites to enhance stability and minimize experimental variation. A similar accuracy between training and LOOCV in our training cohort (AUC = 0.94 vs. 0.91) suggested a minimal risk of overfitting (Extended Data Fig. 9a). We then applied the model to the two independent validation cohorts that included 30 CRC patients and 8 controls in validation cohort 1 and 7 CRC patients and 8 controls in validation cohort 2 (Supplementary Table 4–5, Extended Data Fig. 9b), and observed similar results (AUC = 0.89 and 0.93, respectively, Fig. 2h and Extended Data Fig. 9c). Notably, in validation cohort 1, which included 11 adenoma patients and 8 stage I CRC patients, the model effectively distinguished both adenoma and stage I CRC from non-cancer controls (Extended Data Fig. 9d–e). Despite inter-individual variability, the identified cancer-associated microbial methylation patterns remained broadly consistent across cohorts, supporting the clinical potential of these biomarkers (Extended Data Fig. 9c). We further applied our strategy to pancreatic cancer (PANC), a cancer that may not directly associate with microbiota. In a small cohort (7 PANC patients and 8 non-cancer controls), microbiome-derived cfRNA methylation profiles also showed differences between cancer patients and non-cancer controls (Extended Data Fig. 10 and Supplementary Table 6). While this result hints a broader applicability beyond CRC, further validation in larger cohorts and mechanistic understanding are needed in the future.

In summary, we demonstrate that methylation levels in microbiome-derived cfRNA are effective and promising biomarkers for CRC diagnosis, offering a significant advantage over microbial abundance profiles of cfRNA or cfDNA. Unlike abundance-based approaches, methylation changes in microbiome-derived cfRNA may reflect the intrinsic status and activity of the microbiota, making them more sensitive to cancerous microenvironment^37^. Our approach achieved high accuracy in CRC detection, particularly in the earliest stages of the disease. Additionally, host RNA modification level changes in tissues or plasma may also hold promises for disease diagnosis or prognosis. Our findings highlight the potential of cfRNA and their modification patterns as reliable biomarkers for monitoring host microbiome dynamics, not only for cancer diagnosis but also for other health-related applications. 

## Methods

### Patient recruitment

A total of 27 patients with colorectal cancer and a total of 36 sex, age matched non-cancer individuals were recruited from the Lutheran General Hospital and the University of Chicago Medical Center. Blood from patients with CRC was collected before surgical resection (CRC). Peripheral blood samples from all participants were collected using standardized venipuncture protocols in antecubital venipuncture regions, with skin surfaces disinfected with 70% ethanol prior to phlebotomy to ensure aseptic conditions. All specimens were visually inspected to confirm absence of hemolysis. Blood from non-cancer individuals was collected from those undergoing screening colonoscopies at the University of Chicago Medical Center, with strict adherence to the same pre-analytical quality control measures, and only included subjects with no tumor upon post-colonoscopy pathological analyses.

We included two validation cohorts in this study. Validation cohort 1 consisted of 8 non-cancer individuals and 30 patients with CRC. These samples were obtained from stored specimens, which were collected and processed by a separate research group at the same institute. To ensure comparability, CRC patients at different disease stages were age- and sex-matched with the non-cancer individuals. Validation cohort 2 included samples from additional individuals, with 8 non-cancer individuals and 7 patients with CRC, who were recruited in a different phase of this study. These samples were collected using the same procedures as the training cohort and processed by the same research group.

### Inclusion & ethics statement

All experimental methods are in full compliance with the Helsinki Declaration. The informed consent form was obtained from each study participant before the study. All patients were consented under the protocol of IRB-10–209A, which was approved by the University of Chicago Institutional Review Board.

### Whole blood processing

For all cohorts, ~ 3 ml of whole blood samples was collected in K2 EDTA or Streck vacutainers. Plasma separation was performed within 4 hours of whole blood collection. Blood samples were first centrifuged at 1,350×g for 12 min at 4°C, to collect the upper plasma layer. The plasma layer was then transferred to a clean 15 ml tube and centrifuged again at 1,350×g for 12 min at 4°C again. Later, the upper plasma layer was transferred to a clean 1.5-ml EP tube and further centrifuged at 13,500×g for 5 min at 4°C to ensure complete removal of cell debris. The harvested plasma was split into 600 μl aliquots and stored at −80 °C until cfRNA isolation.

### cfRNA extraction from plasma samples

cfRNAs were isolated from 600 μl plasma using QIAamp ccfDNA/RNA Kit (Qiagen, Cat. 55184) according to the manufacturer’s protocol. In brief, 180 μl RPL buffer was added into 600 μl plasma, followed by vortexing for 5 s. After leaving at room temperature for 3 mins, 100 μl RPP buffer was added. Then, the EP tube cap was closed, and immediately mix vigorously by vortexing 10 s for 2 times, which is important to disperse the solid. Incubate the EP tube on ice for 3 min. After the standard washing and concentration steps following the manufacturer’s protocol, the purified cfRNA was finally eluted in 14 μl of RNase-free water.

### Cell culture

HepG2 cell line was purchased from the American Type Culture Collection (ATCC) and maintained in DMEM (Gibco, 11995) with 10% FBS and 1% penicillin/streptomycin. Cells were cultured at 37 °C with 5.0% CO_2_ in a Heracell VIOS 160i incubator (Thermo Scientific).

### Culture of the *Stenotrophomonas maltophilia*

*Stenotrophomonas maltophilia* strains were obtained from the clinical microbiology laboratory after confirming the strend and subsequently cultured in Eugon Broth medium. Bacterial growth was carried out under aerobic conditions at 37°C with continuous agitation (180 rpm) for 24-48 hours.

### Skin specimens

Skin samples were collected from the 2×2 cm^2^ antecubital venipuncture regions of two non-cancer adult donors using sterile scraping technique (30-second scraping duration per site). Following collection, samples were immediately homogenized in 1 mL TRIzol reagent (Invitrogen). The resulting lysates were stored at −80°C until RNA extraction.

### CRC tumor tissues and NATs.

CRC tumor tissues and matched normal adjacent tissues (NATs) were obtained from surgical resections. Tissue samples were immediately snap-frozen in liquid nitrogen and stored at −80°C until processing. For RNA extraction, frozen tissues samples were mechanically homogenized using a stainless-steel bead milling system. Then, TRIzol reagent (Invitrogen) was added into the homogenate and incubated at room temperature for 5 min to ensure complete dissociation of nucleoprotein complexes, followed by centrifugation at 12,000 x g for 10 min at 4°C to remove insoluble debris. The supernatant was transferred to fresh RNase-free tubes for subsequent RNA isolation.

### Small RNA isolation

Total cellular total RNA of HepG2 was isolated with TRIzol reagent (Invitrogen) following the manufacturer’s standard protocol based on isopropanol precipitation. The small RNA fraction (size < 200 nt) was further extracted from the purified total RNA using the mirVana miRNA Isolation Kit (AM1560, Invitrogen), following the manufacturer’s standard protocol.

### LIME-seq library preparation

Plasma cell-free RNA (cfRNA) or small RNA from HepG2 (<200 nt) were fragmented to lengths of ~35-55 nucleotides using the 10X RNA Fragmentation Reagent (AM8740, Invitrogen), which was used as 15X with heating at 70 °C for 14 mins. Purify the fragmented RNA using the Oligo Clean & Concentrator kit (Zymo Research) with a final elution volume of 10 μl. The subsequent 3′-end repair involved the preparation of a mixture of fragmented RNA, 2 μl of 10× T4 Polynucleotide Kinase Reaction Buffer (B0201S, NEB), 2 μl of T4 PNK, 1 μl SUPERase•In RNase Inhibitor and a proper volume of RNase-free water. Mix the 20 μl reaction mixture very well and incubate at 37°C for 1 hour. 3’-end-repaired RNA fragments were purified by Oligo Clean & Concentrator kit and eluted in 10 μl of RNase-free water.

For 3′-adapter ligation, RNA fragments were denatured with 1ul of 10 μM RNA 3′-linker (5′rApp-NNNNN AGATCGGAAGAGCGTCGTG-3SpC3, synthesized by IDT) by heating at 70°C for 2 minutes and then immediately moved onto ice. Then a mixture of 2.5 μl of 10× T4 RNA Ligase Reaction Buffer (NEB), 7.5 μl of 50% PEG8000, 1 μl SUPERase•In, and 2 μl T4 RNA ligase 2 truncated KQ (NEB) was prepared, followed by mixing well and incubating at 25°C for 2 hrs and then at 16°C for 10 hrs. The excessive adapters were removed by adding 2 μl of 5′-deadenylase (NEB) with an incubation at 30 °C for 45 mins, followed by adding 1.0 μl of RecJf (NEB) with an incubation at 37 °C for 45 mins. The 3’-ligated RNA was then purified by RNA Clean & Concentrator (Zymo Research) and eluted in 10 μl of RNase-free water.

Reverse transcription was initiated by mixing the 3’-ligated RNA with 1 ul of 2.0 μM RT primer (5′-ACACGACGCTCTTCCGATCT-3′, synthesized by IDT) and heating at 65°C for 2 minutes. Then move the samples onto ice immediately. A mixture of 2 μl of 10× AMV Reverse Transcriptase Reaction Buffer (NEB), 2 μl of 10 mM dNTPs, 1 μl RNaseOUT (Thermo Fisher Scientific), 2 μl HIV Recombinant Reverse Transcriptase (Worthington) and a proper volume of RNase-free water was prepared, to give a final volume of 20 μl for a one-hour incubation at 37°C. Upon the completion of RT, 1.0 μl volume of RNase H (NEB, M0297L) was added to the reaction mixture, followed by incubation at 37 °C for 20 min and 70 °C for 5 min.

The produced cDNAs after RT were purified by Oligo Clean & Concentrator (Zymo Research) and eluted in 10 μl of RNase-free water. A mixture of eluted cDNA and 2.0 μl of 10 μM cDNA 3′-linker (5′Phos-NNNNNAGATCGGAAGAGCACACGTCTG-3SpC3, synthesized by IDT) was denatured at 75 °C for 2 min and then moved onto ice immediately. 3-μl of 10× T4 RNA Ligase Reaction Buffer (NEB), 15 μl of 50% PEG8000, 3 μl of 10 mM ATP, and 1.0 μl of T4 RNA ligase 1 (high concentration, NEB) were added to the cDNA–adapter mixture and mixed very well. Incubate the ligation reaction mixture at 25 °C for 12 h. The libraries were amplified using universal and indexed primers from NEBNext Multiplex Oligos for Illumina (NEB). All libraries were sequenced on an Illumina NovaSeq 6000 or NovaSeqX with SE100 bp mode.

The LIME-seq method enables high mutation ratio detection across multiple RNA modifications, including m^1^A, m^3^C, m^1^G, m^2^_2_G, m^3^U, etc., with an enhanced read-through at these methylation sites. Such performance has been facilitated by the robust HIV RT enzyme, and it effectively uncovered methylation fraction information at methylated sites.

### Raw reads processing and mapping on human genome

The sequencing data were all trimmed with the cutadapt tool (version 4.6)^57^ to remove adapters and low-quality reads (length shorter than 28 bp). Then, PCR duplicates were removed with the BBMap tool (39.10, https://sourceforge.net/projects/bbmap/). Later, random barcodes at reads end were trimmed, and low-quality reads were removed using the cutadapt tool again. Then the remaining reads were aligned to the human genome (GRCh38) using STAR (version 2.7.11a) ^58^ and bowtie2 (version 2.5.1)^59^ allowing a maximum of three mismatches. We compared the cfRNA mapping results between bowtie2 and STAR, mis-splicing and short reads in the libraries promote us to use bowtie2. Based on the aligned results from bowtie2, we normalized the data by calculating the reads per million (RPM) and performed student t-test (two tailed) to calculate the *p* value for the differential expression. Most differentially expressed protein-coding transcripts identified by LIME-seq are miRNAs occurring within these transcripts. For analyzing cfRNA transcripts from the human genome, we set a minimum RPM of 20 in at least one group (CRC or non-cancer), focused on non-protein coding transcripts, and excluded redundant transcripts.

### Access the stability of the biomarkers

To evaluate the stability of the mutation signature of microbiome derived cfRNA biomarkers, three blood tubes were collected from each of two healthy donors under standardized conditions. Immediately after collection, all tubes were placed on ice and transferred to a 4°C refrigerator for temporary storage prior to plasma isolation. The experimental design incorporated three processing timepoints: plasma isolation initiated within 2 hours, plasma isolation after 8-hour refrigeration, and plasma isolation after 24-hour refrigeration. Plasma was separated through our standard protocol and stored in 80°C after extraction.

### Visualization of reads coverage

After aligning sequences to the reference genome, we employ “*depth*” function in the samtools (version 1.16.1)^60^ to extract regions corresponding to various RNA species. Following the extraction of read depths at these sites, we utilize an in-house Python pipeline to plot the read coverage across these regions.

### Quantification of the methylation in HepG2 cell, tissues and host part in cfRNA

After mapping to the human genome with bowtie2, the generated .bam files were split into positive and negative strands and sorted using samtools. Sequence variants were identified by measuring the base composition at each position using fine-tuned bam-readcount (https://github.com/genome/bam-readcount). The generated bam-readcount output results were parsed and analysed to calculate the misincorporation ratio at each methylated site (m^1^A, m^1^G, m^2^_2_G and m^3^C) in tRNA, followed by confirmation using direct visualization through IGV software (https://software.broadinstitute.org/software/igv/). For quality control of cfRNA, we established a criterion where the average mutation ratio at the m^1^A sites within human tRNA must exceed 0.6. All samples used in this study passed this cut-off.

### Quantification of the microbal abundance and methylation in microbiome from cfRNA

Cleaned reads that failed to align to the human genome were applied using Kraken2 (version 2.1.3)^61^with its standard database, which contains bacterial, archaeal, viral, and human sequences. Based on the aligned results from Kraken2, we normalized the data by calculating the reads per million (RPM). Microbial species were retained if the median read count exceeded 10 in at least one group (CRC or non-cancer). We plotted differentially expressed microbes with RPM greater than 20 for comparison with host transcripts.

To refine methylation analyses, we extracted reference genomes for the identified species from the Kraken2 database. To reduce redundancy and improve mapping specificity, a streamlined microbial reference was generated by selecting up to ten representative genomes per species. Subsequently, we aligned the cleaned reads that failed to align to the human genome to this curated microbiome reference genome using bowtie2. The alignments generated by bowtie2 were applied in the calculation of the microbiome mapping ratio. To assess the taxonomic resolution of short cfRNA sequences, we performd BLAST-based validation. Our tests suggested that short reads from cfRNA generally achieve reliable mapping at the order level, while tRNA-derived sequences provide sufficient resolution to support taxonomic classification at the genus or family level. Based on this, we report microbial identities at the genus or family level in this study.

Variant identification within these sequences is then achieved through the precise analysis of base composition at each genomic position, employing bam-readcount tool (1.0.1, https://github.com/genome/bam-readcount). The generated bam-readcount output results were parsed and analyzed to calculate the misincorporation ratio.

### Analysis of the methylation sites in microbiome

To ascertain whether a site’s mutation signal is due to methylation or a single nucleotide polymorphism (SNP), we examined the mutation signals induced by HIV-RT. Our analysis indicates that signals attributed to methylation typically result from a combination of mutations, whereas SNPs are characterized by a single mutation type (e.g., A>G). Consequently, at any given site, if the mutation ratio for a specific base exceeds 95% of the total mutation ratio, the site is classified as an SNP. Otherwise, this site will be annotated as the methylation.

### Differential methylation analysis in microbiome

We organized raw bam-readcount data and applied filtering to minimize random variation. Specifically, mutational sites in each sample with a depth of less than 3 were deemed insufficient (considered as empty) and excluded from subsequent analyses to ensure robustness in calculating *p* values and the average mutation ratio. The analysis proceeded with the establishment of two critical thresholds: (1) a minimum median depth of more than 5 reads in at least one of the groups (CRC or non-cancer), and (2) an average mutation ratio exceeding 0.10 in at least one group (CRC or non-cancer). These criteria significantly refined the dataset, resulting in approximately 9,000 eligible sites for further analysis. We then assessed these sites for differential methylation using a two-tailed Student’s t-test and visualized the results through heatmap.

### Statistics-based models

Support vector classifier used in this work can be achieved by Scikit-learn (1.5.1)^62^ with python (3.12.3). The misincorporation ratio of a subgroup of 330 methylation sites have been used as the input for the model. These data are then partitioned into combined training and testing sets. Within the model, any missing feature values are imputed with the feature’s average value across samples, ensuring completeness. To evaluate the model’s performance, we apply several validation techniques, including LOOCV, 4-fold Cross-Validation, and bootstrapping, the latter using 25% of the data as a test set.

### Explanation of the statistics-based models

SHAP values were calculated to estimate feature attribution for each endpoint and model individually^63^. SHAP values integrate Shapley values—rooted in cooperative game theory—to compute the average incremental effect of each feature on the model’s prediction, adhering to the principle of local additivity. A key attribute of Shapley values is their ability to quantify the contribution of each player (or feature) in a game (or model) by comparing the outcome with all players (or features) involved versus an absence of players (or features). In the context of statistical models, this translates to SHAP values summing to the difference between the expected model output (baseline) and the actual output for a specific prediction. This ensures a comprehensive and fair attribution of each feature’s impact on the prediction outcome. In our work, we apply 4-fold cross-validation in calculating the SHAP values for every feature across all samples.

## Extended Data

**Extended Data Fig. 1 |:**
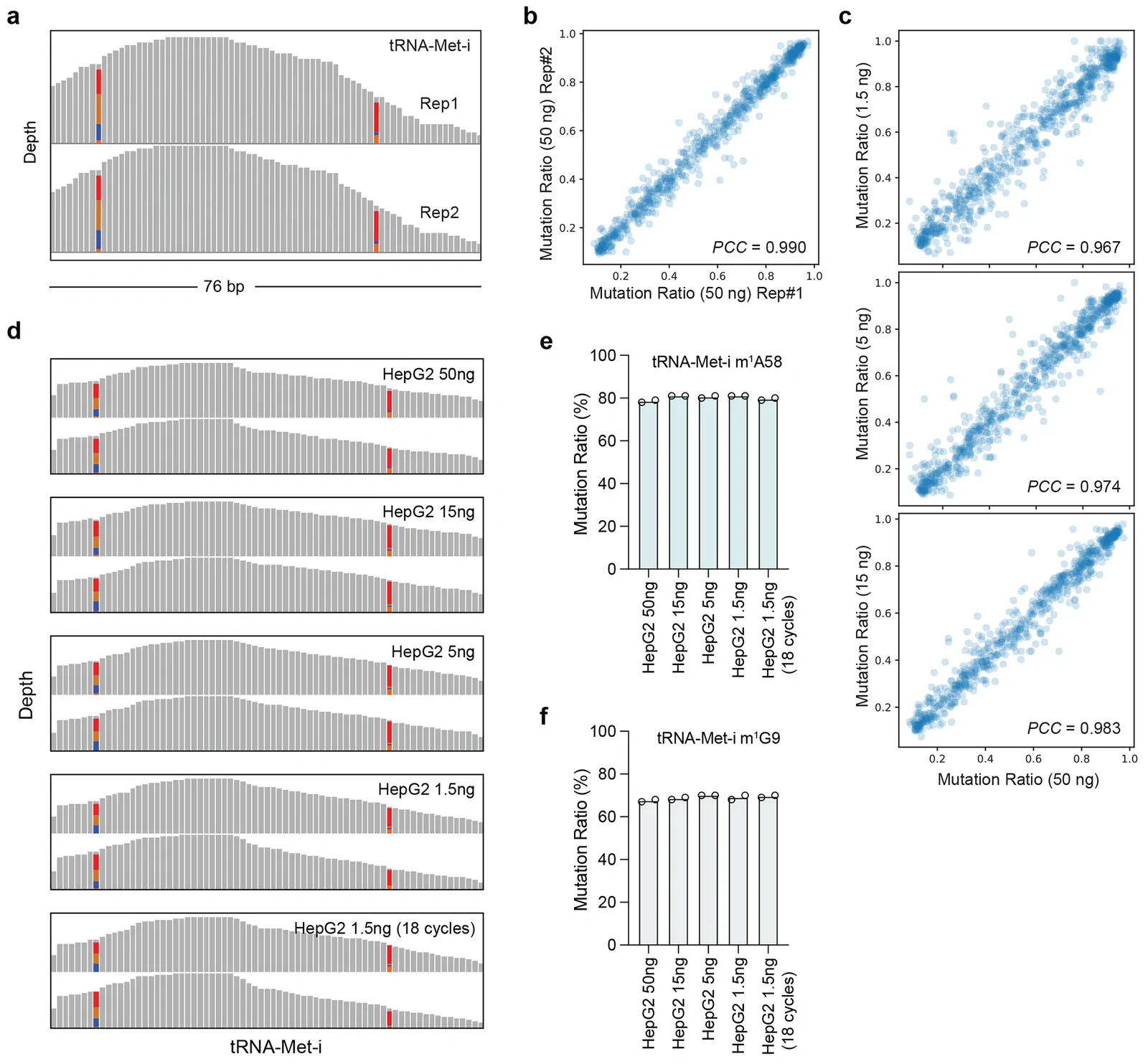
Quantitative and robust identification of RNA methylations in small RNAs from HepG2 cells by LIME-seq. **a**, IGV plot of a representative tRNA-Met-i region in HEPG2 small RNA, displaying the reads coverage and observed mutation sites. **b**, Correlation of mutation ratios between two replicates of HEPG2 small RNA (50 ng) detected by LIME-seq. PCC represents Pearson’s correlation coefficient. **c**, Correlation of mutation ratios detected in HepG2 small RNA across different input amounts (50 ng, 15 ng, 5 ng, and 1.5 ng). **d**, IGV plot of a representative tRNA-Met-i region in HepG2 small RNA with varying input amounts. **e**–**f**. Bar plots showing mutation ratios of RNA methylation sites at m^1^A_58_ (**e**) and m^1^G_9_ (**f**) in tRNA-Met-i across different RNA input amounts. Each data point represents one technical replicate.

**Extended Data Fig. 2 |:**
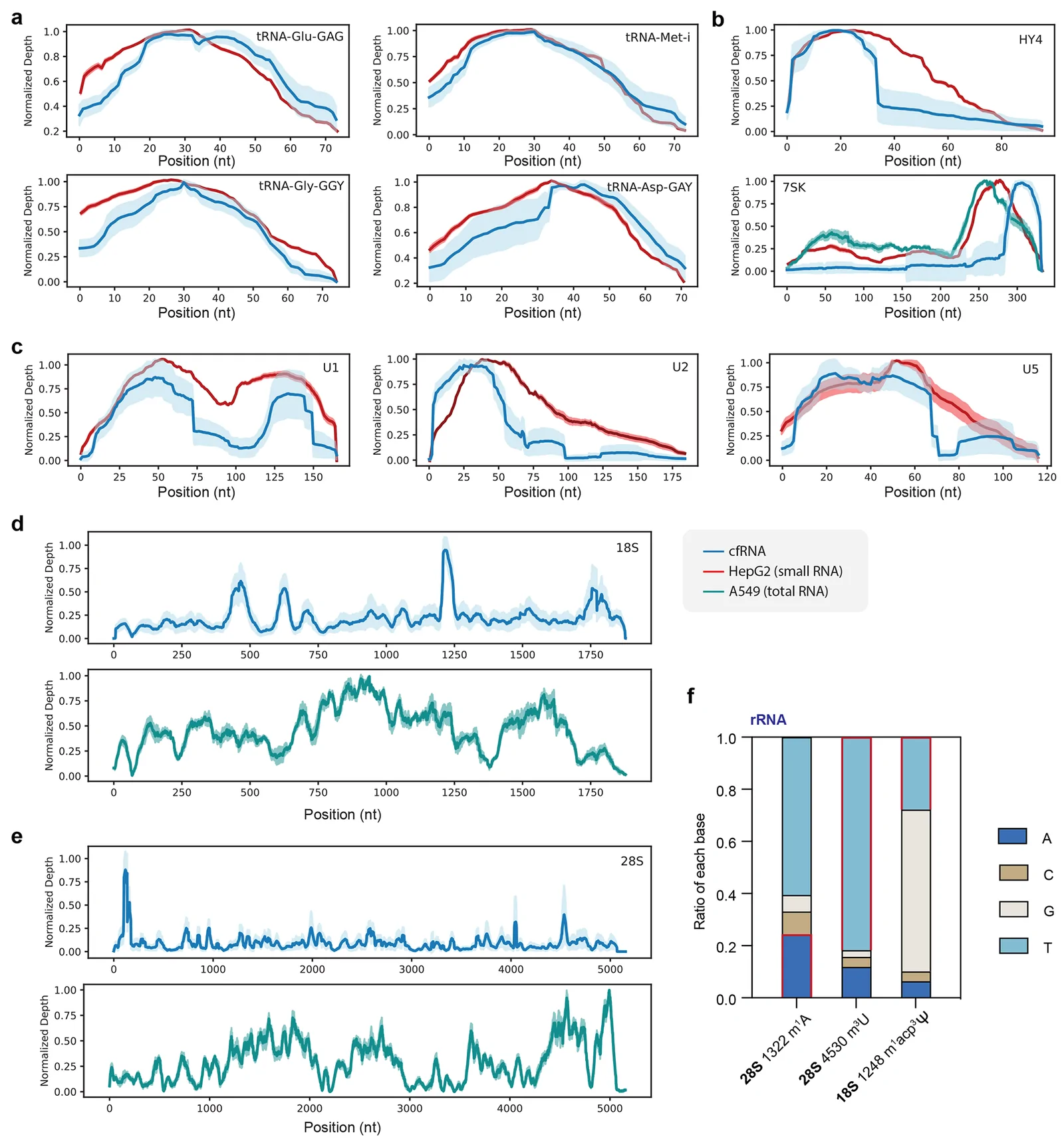
The reads coverage and profile of host RNAs in human plasma cell-free RNAs, revealed by LIME-seq. **a**–**e**, Line plot shows the coverage of cfRNA LIME-seq reads in tRNA (**a**), ncRNA (**b**), snRNA (**c**), 18S rRNA (**d**), and 28S rRNA (**e**) from the human genome. The dark lines represent the average coverage across cfRNAs from 36 non-cancer individuals, while the light regions indicate the standard deviation of RNA coverage variability in these individuals. **f**, Stacked bars show the mutation ratios of methylation sites in rRNAs detected by LIME-seq in cfRNAs.

**Extended Data Fig. 3 |:**
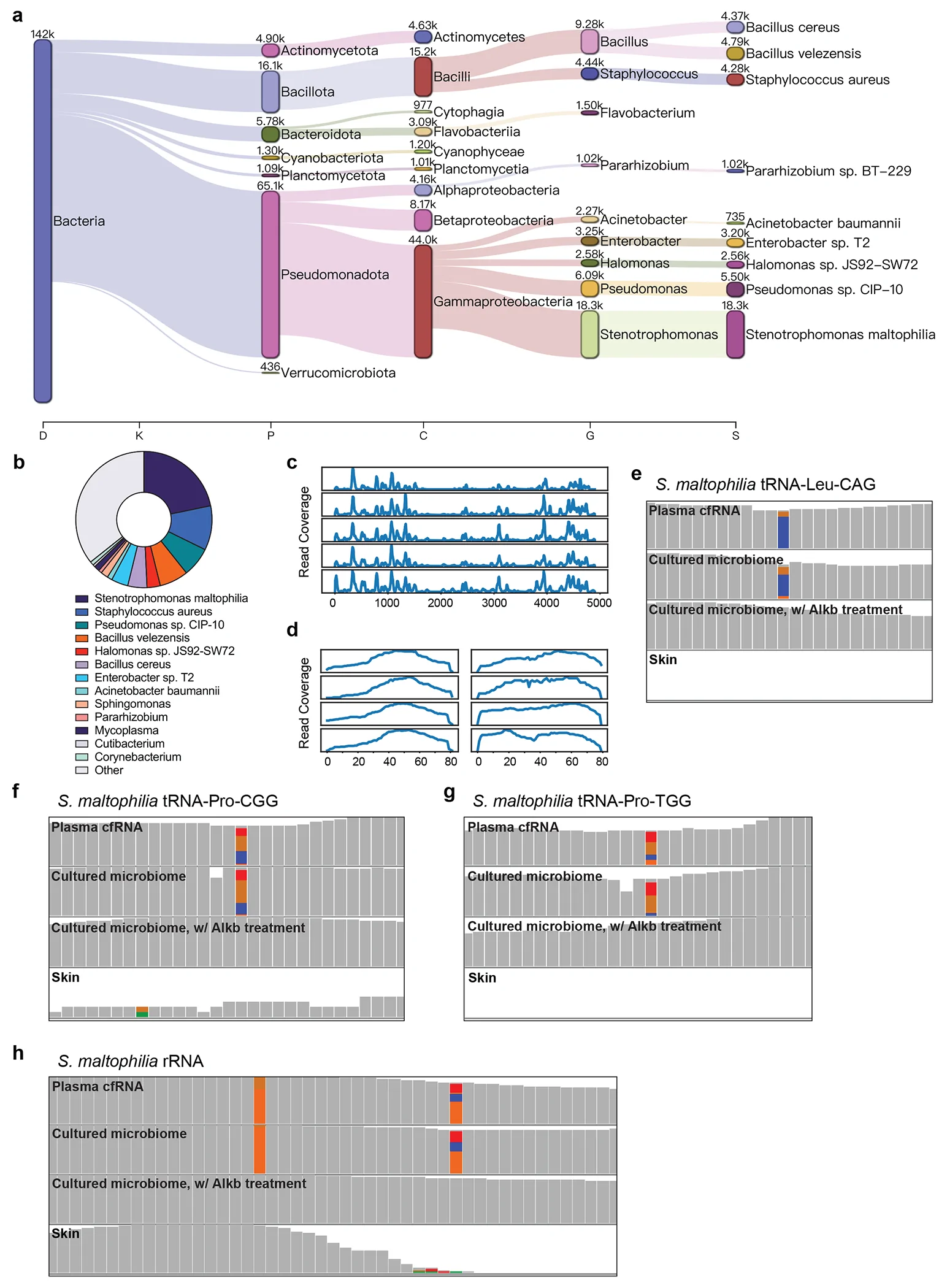
The reads coverage and methylation profile of microbiome-derived cfRNA in human plasma, revealed by LIME-seq. **a**, Hierarchical visualization of the composition and relative abundance of microbial species identified in cfRNA using LIME-seq for one typical health individual. **b**, The pie chart shows the percentage of microbiome-derived cfRNA from the top 13 abundant microbial species and the rest (other), when counting LIME-seq reads mapped to diverse microbial genomes. **c**–**d**, Line plot shows the coverage of microbiome-derived cfRNA LIME-seq reads aligned to *Pseudomonas sp. CIP-10* rRNA (**c**), and tRNA (**d**) regions for typical individuals. **e**–**h**, IGV plots of representative tRNA and rRNA regions, including tRNA-Pro-TGG (**d**), tRNA-Pro-cGG (**e**), tRNA-Leu-CAG (**f**), and rRNA (**g**), comparing RNA reads from lab-cultured *S. maltophilia* with plasma cfRNA reads. Demethylation treatment using AlkB was applied to lab-cultured RNA to confirm modifications. Skin samples were used as a negative control, showing distinct coverage patterns and low abundance reads indicating that these reads were not from *S. maltophilia* but other microbes.

**Extended Data Fig. 4 |:**
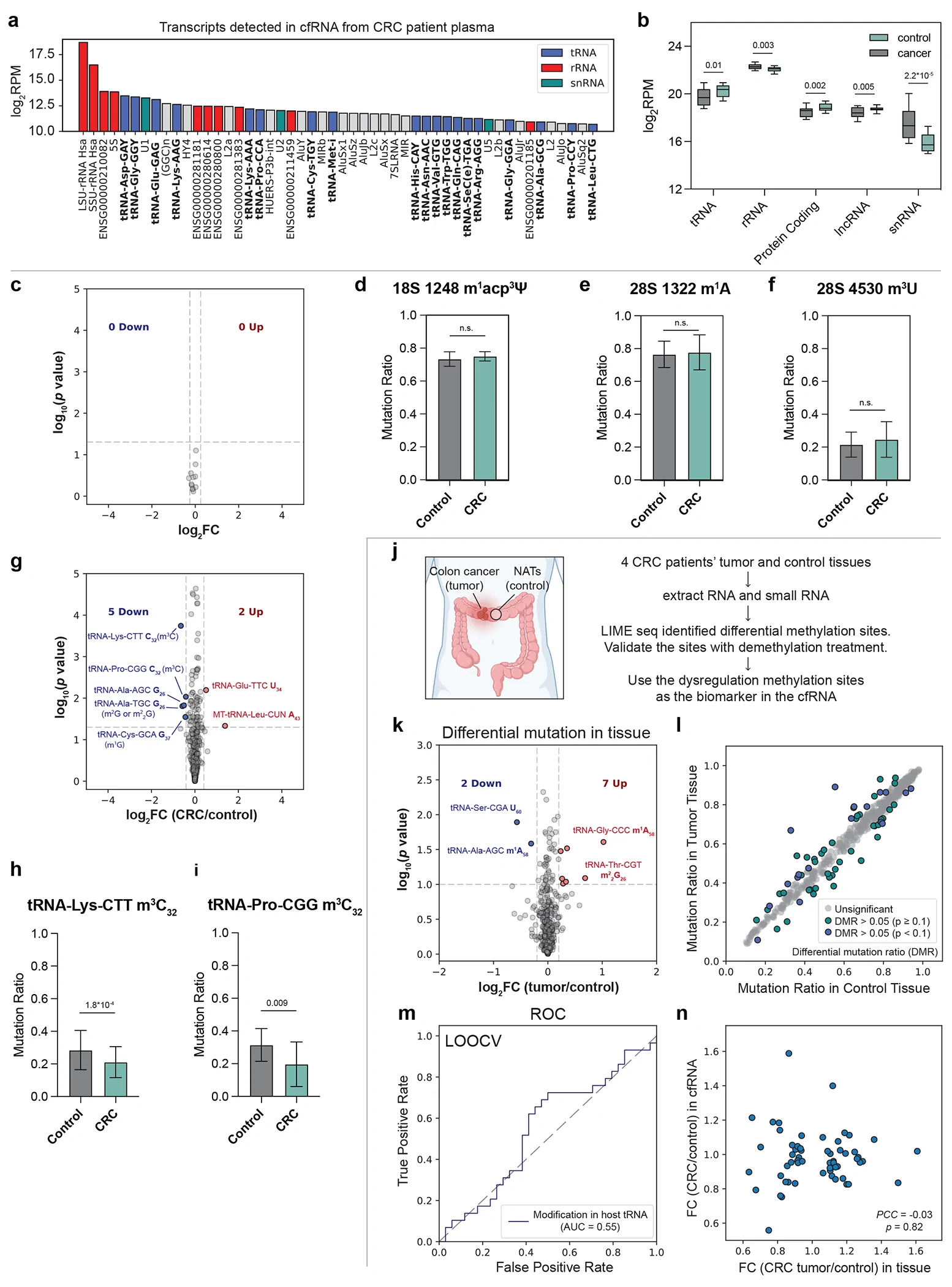
Differential expression and methylation profiles for host RNAs in plasma cfRNA from CRC patients versus non-cancer controls. **a**, Bar plots showing the relative abundance from LIME-seq of the top 50 highly expressed transcripts identified in CRC patient plasma cfRNA. **b**, Box plots comparing the abundance of various human RNA transcripts in CRC patients and non-cancer individuals. *p* value is obtained by student t-test (two tailed). Box plots showing the median and 25th to 75th percentile, and whiskers for the 10th to 90th percentile. **c**, Volcano plot showing differential RNA methylation in human rRNA between CRC patients and non-cancer individuals detected by LIME-seq. *p*-values were calculated using an unpaired t-test (two tailed). **d-f**, Bar plots comparing mutational signatures in human 18S rRNA m^1^acp^3^Ψ (**d**), 28S rRNA m^1^A (**e**), and 28S rRNA m^3^U (**f**). Data are presented as mean values +/− SD. **g**, Volcano plot showing differential RNA methylation in human tRNA between CRC patients and non-cancer individuals, with upregulated sites (red, *n* = 2) and downregulated sites (blue, *n* = 5) highlighted. *p* values were calculated using an unpaired t-test (two tailed). **h-i**, Box plots comparing mutational signatures in human (**h**) m^3^C_32_ at tRNA-Lys (CTT), (**i**) m^3^C_32_ at tRNA-Pro (CGG). *p* values obtained by student t-test (two tailed). Data are presented as mean values +/− SD. **j**, Schematic of the pipeline for identifying differential tRNA methylation sites in CRC tumor tissues versus normal adjacent tissues (NATs) from four CRC patients. **k–l**, Volcano plot (**k**) and scatter plot (**l**) showing differential human tRNA methylation between CRC tumor tissues and NATs. *p* values were calculated using a paired t-test. **m**, ROC curve for a classifier based on mutational signatures of human tRNA in cfRNA, using tRNA site mutation ratios as input, validated by LOOCV (**I**). **n**, Scatter plot showing low correlation in mutation ratio fold changes between CRC tumors versus NATs and cfRNA from CRC patients versus non-cancer individuals. Data are presented as mean ± SD in **b, d, e, f, h**, and **i**, *n* = 36 for control group and *n* = 27 individuals for CRC group.

**Extended Data Fig. 5 |:**
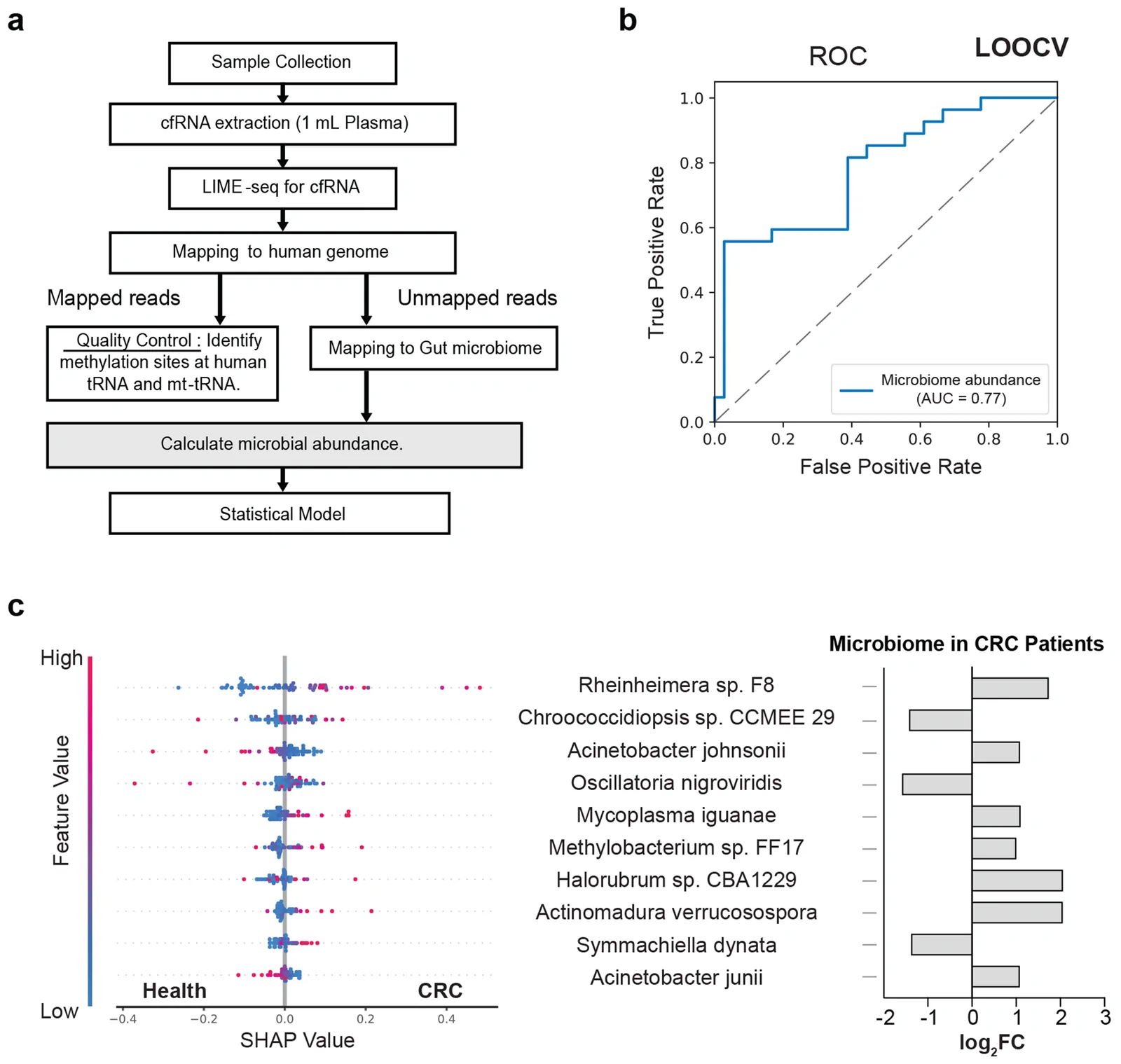
LIME-seq reveals the differential abundance of microbial species as the biomarker for CRC diagnosis. **a**, Workflow for the CRC diagnosis based on the abundance of microbial species in human plasma as revealed by LIME-seq. **b**, ROC curve of the classifier based on microbial species abundance obtained from LIME-seq comparing CRC patients and non-cancer individuals, validated using LOOCV. **c**, The SHAP values from the classifier model reveal the important microbe in CRC (*p* value obtained by t-test between non-cancer individuals and CRC patients is marked in blue). The SHAP values illustrate the significance of different inputs in determining the predictive capability of the statistical model.

**Extended Data Fig. 6 |:**
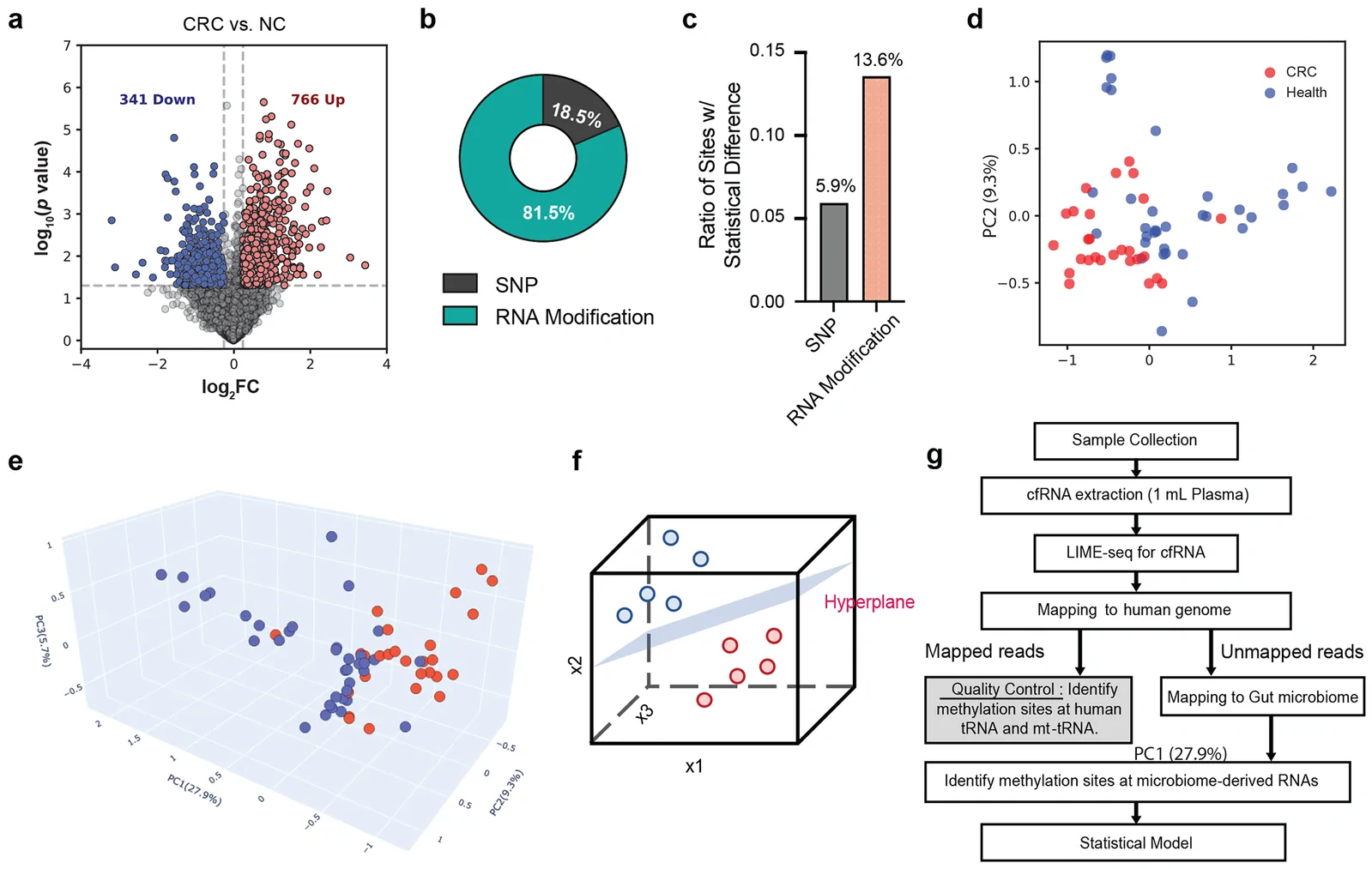
RNA modification profiles in microbiome-derived cfRNA distinguishes CRC patients from non-cancer controls. **a**, Volcano plot shows the differential RNA methylation analysis in microbiome-derived cfRNA, comparing mutation signatures between CRC patients and non-cancer individuals. *p*-values were calculated using an unpaired t-test (two tailed). **b**, The pie chart shows the composition of mutational signature accessed by LIME-seq in microbiome-derived cfRNA. The percentage of SNP and RNA modifications is shown in figure. As the mutation patterns from HIV-RT shown in Extended Data Fig. 3, RNA modifications can be detected as mixed mutational signature during reverse transcription process by HIV-RT. **c**, Bar plot shows the ratio of mutational signature with statistical difference for both SNP and modification sites. A much higher ratio in modification sites highlight its dynamic between CRC patients and non-cancer individuals. **d**, Two-dimensional principal component analysis (PCA) on the mutation signature of significantly methylated sites in cfRNA derived from the microbiome in colorectal cancer patients and non-cancer controls. **e**, Three-dimensional PCA of the mutation signature at significantly methylated sites in cfRNA from the microbiome of CRC patients and non-cancer controls suggests that higher dimensionality enhances classification accuracy between CRC patients and non-cancer individuals compared to two-dimensional PCA. **f**, Schematic overview of the hyperplane in SVC. **g**, Workflow for developing a diagnostic model for colorectal cancer patients using epitranscriptional signatures in microbiome-derived cfRNA with LIME-seq.

**Extended Data Fig. 7 |:**
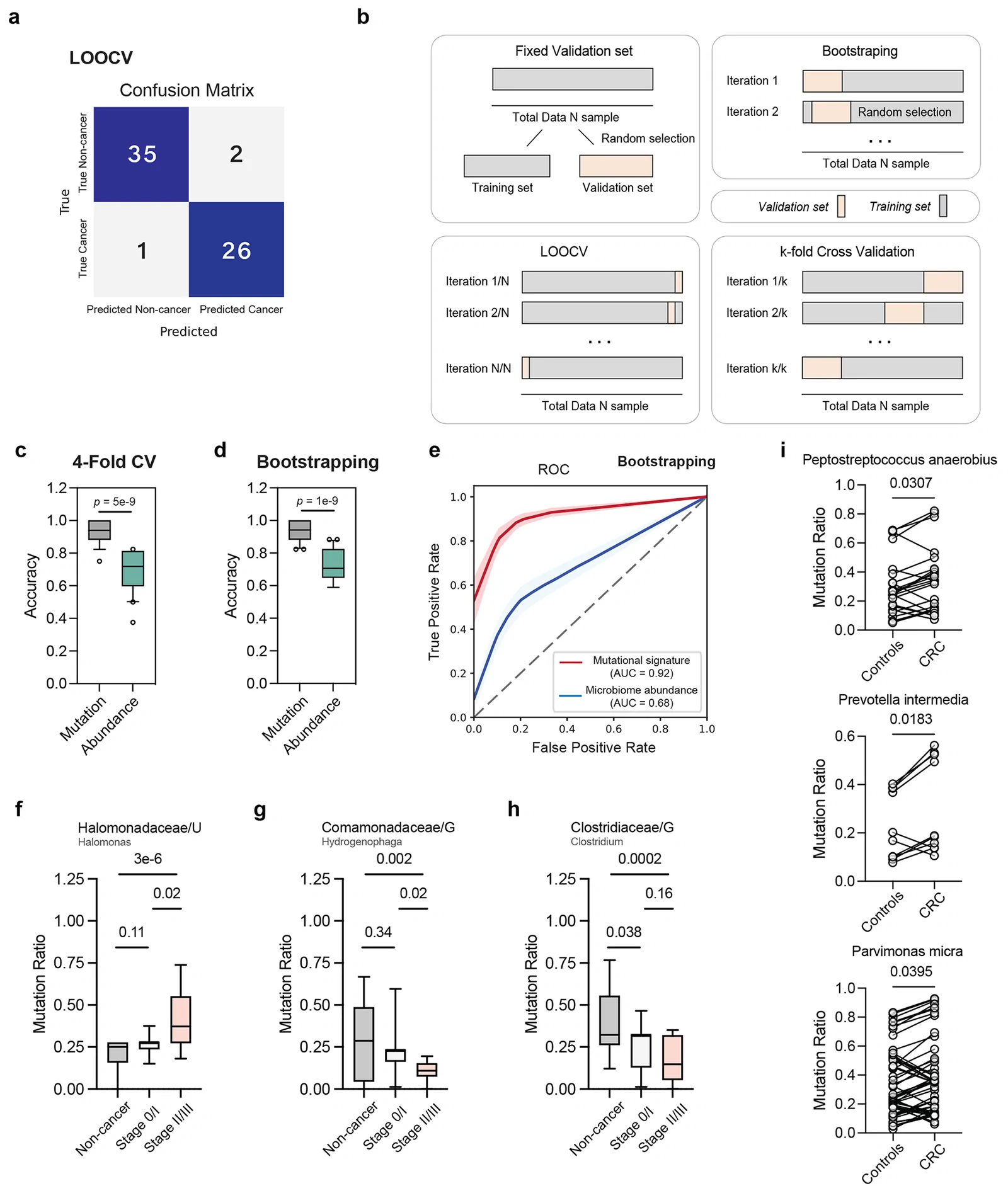
Predictive power and biological stratification of microbiome-derived cfRNA modifications in CRC diagnosis. **a**, Confusion matrix of the classifier based on mutational signature from LIME-seq between CRC patients and non-cancer controls, evaluated through LOOCV. **b**, Schematic overview of different validation methods. **c**, The prediction accuracy of the classifier for CRC diagnosis with 4-Fold cross validation. The error bar comes from the five repeats of 4-fold cross validation (n = 20, *p* = 5*10^’9^, obtained by student t-test (two tailed)). **d**, The prediction accuracy of the classifier for CRC diagnosis with bootstrapping with 25% dataset as the validation set. The error bar comes from the 20 repeats (n = 20, *p* = 1*10^’9^, obtained by two tailed student t-test (two tailed)). Box plots showing the median, 25th–75th percentiles (box), and 10th–90th percentiles (whiskers). **e**, ROC of the classifier based on mutational signature and abundance of microbial species from LIME-seq between CRC patients and non-cancer controls, evaluated through bootstrapping with 25% dataset as the validation set. The light color regions reflect the mean values +/− standard error (SEM) of 20 repeats. **f–h**, Dynamic mutational sites in (**f**) *Halomonadaceae*, (**g**) *Comamonadaceae*, (**h**) *Clostridiaceae* across different stages identified by the statistical model. *p* value obtained by student t-test (two tailed) is shown in the figure. n = 36 (control), n = 11 (stage 0/I), and n = 16 (stage II/III). Box plots show the median, 25th–75th percentiles (box), and 10th–90th percentiles (whiskers). **i**, Mutational profiles of differential mutation sites observed in *Peptostreptococcus anaerobiusm, Prevotella Intermedia*, and *Parvimonas micra*. Each point represents the group-average methylation level across differential sites from 36 controls or 27 CRC patients. *p*-values were calculated using a paired t-test.

**Extended Data Fig. 8 |:**
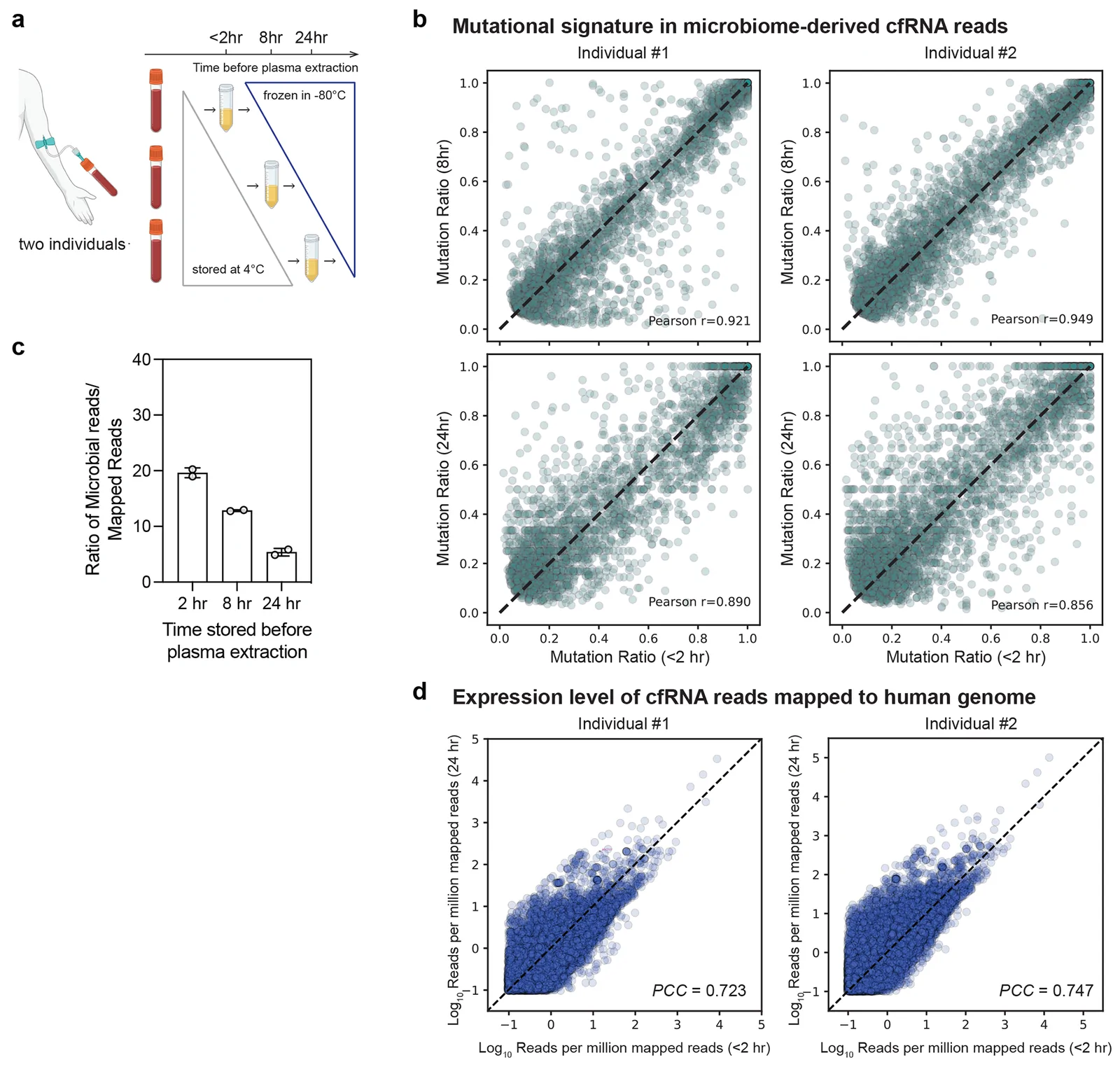
Evaluating the stability of the mutational signatures detected in microbiome-derived cfRNA. **a**, Schematic of the experimental design for evaluating cfRNA stability. Whole blood was collected from two individuals, with three tubes per individual, and stored at 4°C for <2 hours, 8 hours, and 24 hours before plasma extraction. Plasma was immediately frozen at −80°C after extraction. **b**, Correlation of mutation ratios detected in cfRNA from plasma stored at 4°C for <2 hours, 8 hours, and 24 hours. **c**, Relative proportion of microbial reads observed in cfRNA with different storage conditions. Each data point represents an individual. Data are presented as mean values +/− SD. **d**, Low correlation of host transcript expression levels between cfRNA obtained at 2 hours and 24 hours, suggesting leakage of host cell RNA over time.

**Extended Data Fig. 9 |:**
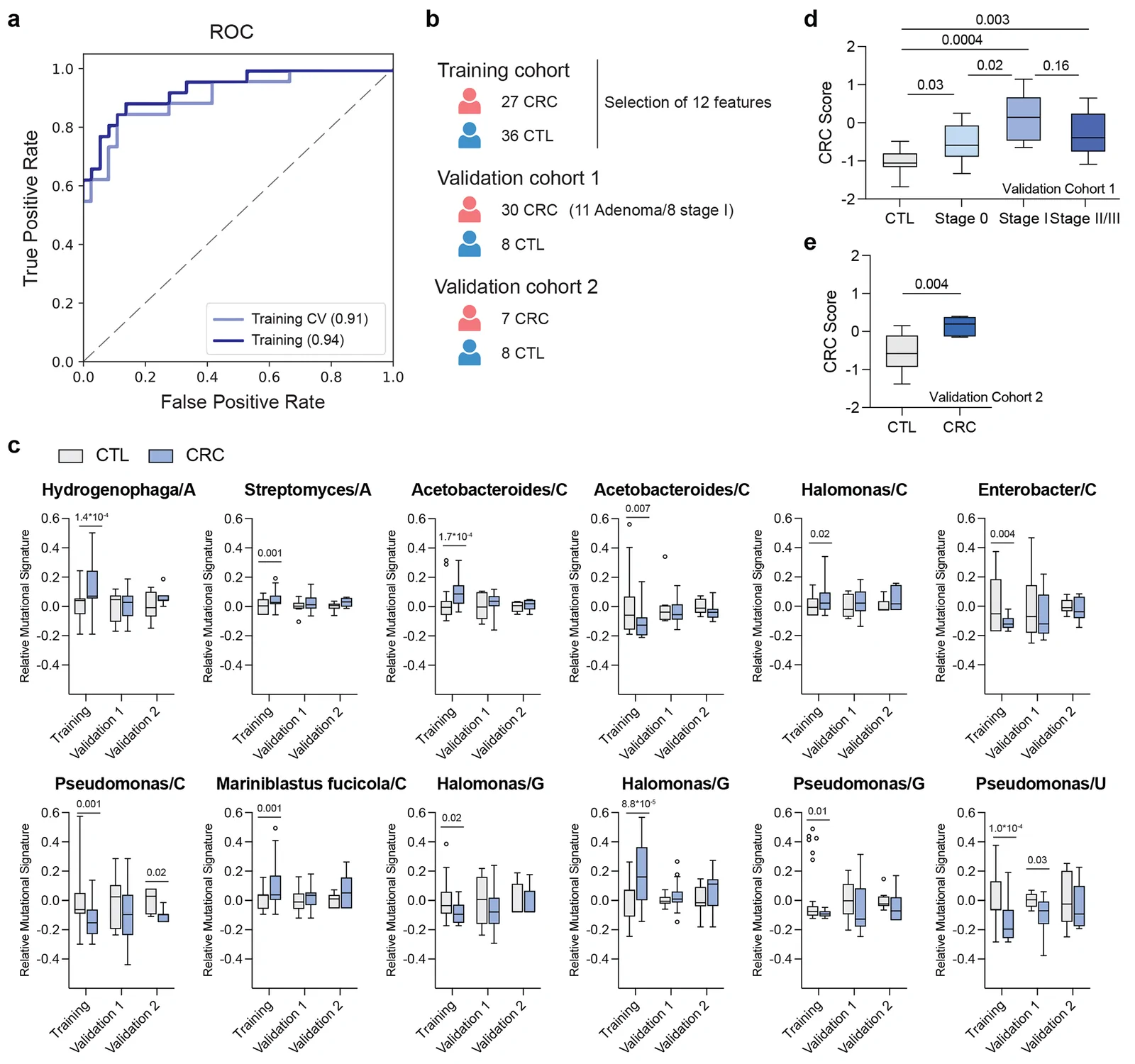
The validation of a subset of mutational signatures as biomarkers for CRC early diagnosis using two external cohorts. **a**, ROC curve of the classifier based on the mutational signature of microbe detected by LIME-seq in the training cohort, evaluated using model fitting and LOOCV. **b**, Schematic of the experimental design and sample sizes for the external validation cohorts. **c**, Mutational signatures of 12 selected sites with normalized, centered mutational level across the training and validation cohorts. Box plots showing the median (center line), the 25th and 75th percentiles (box bounds), and the whiskers extend to data points within 1.5× the interquartile range (Tukey method). Outliers beyond this range are shown individually. **d–e**, Relative CRC scores of patients in external validation cohort 1 (**d**) and cohort 2 (**e**), based on the model with 12 selected sites shown in **c**. Box plots showing the median (center line) and interquartile range (box). Whiskers extend to the minimum and maximum values. Sample sizes (n) for each group are indicated in panel b. *p*-values were calculated using an unpaired t-test (two tailed).

**Extended Data Fig. 10 |:**
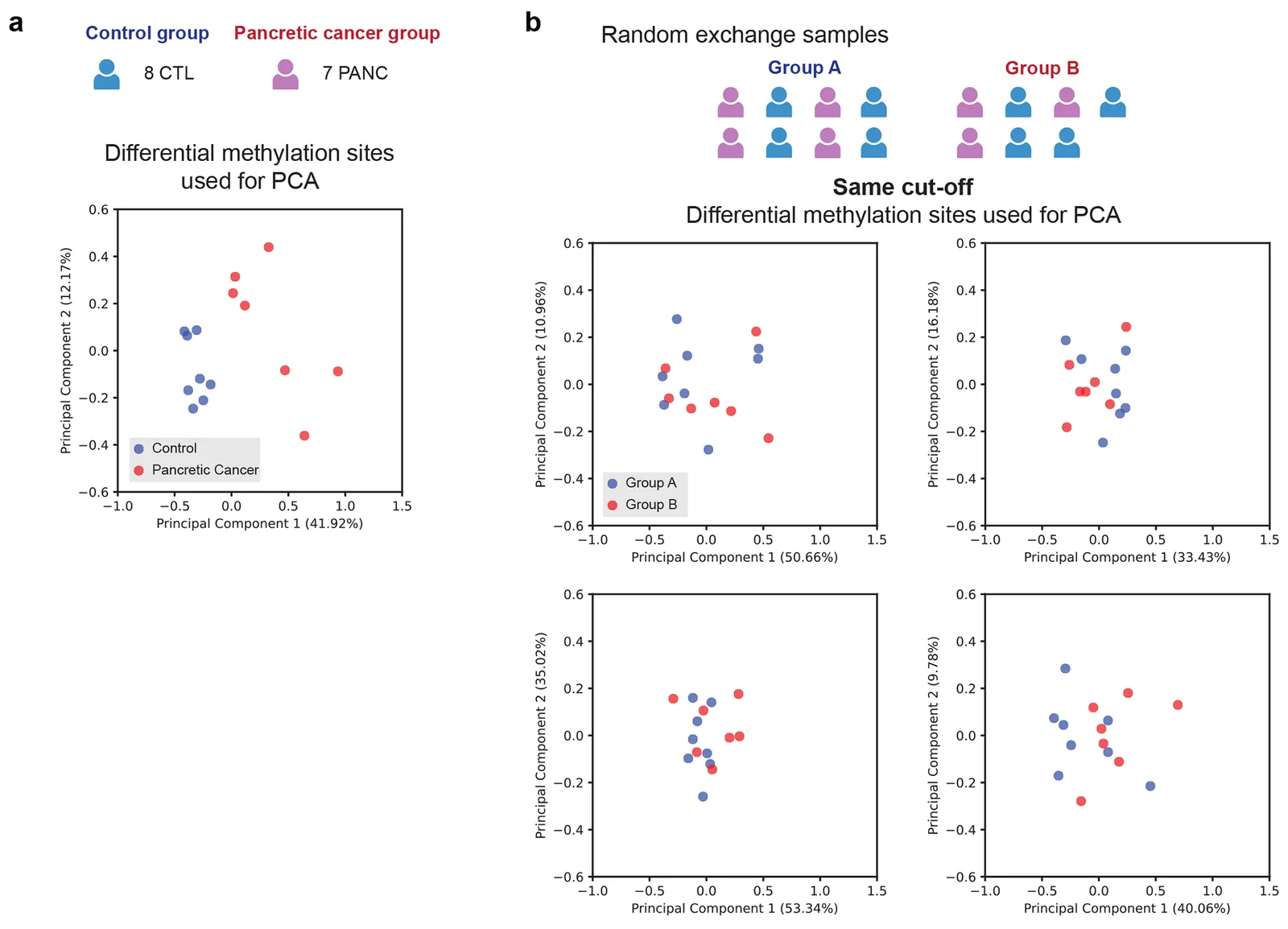
RNA modification in plasma cfRNA might be clinically informative for pancreatic cancer diagnosis. **a**, Two-dimensional PCA illustrating differences in the mutational signatures of differentially methylated sites in microbiome-derived cfRNA between pancreatic cancer patients and non-cancer controls. **b**, Random permutation of patient and control labels significantly reduces the classification ability observed in the PCA.

## Supplementary Material

Supplementary Table

source_data_Figures

Supplemantary Data Fig

source_data_Extended_data

## Figures and Tables

**Fig. 1 |:**
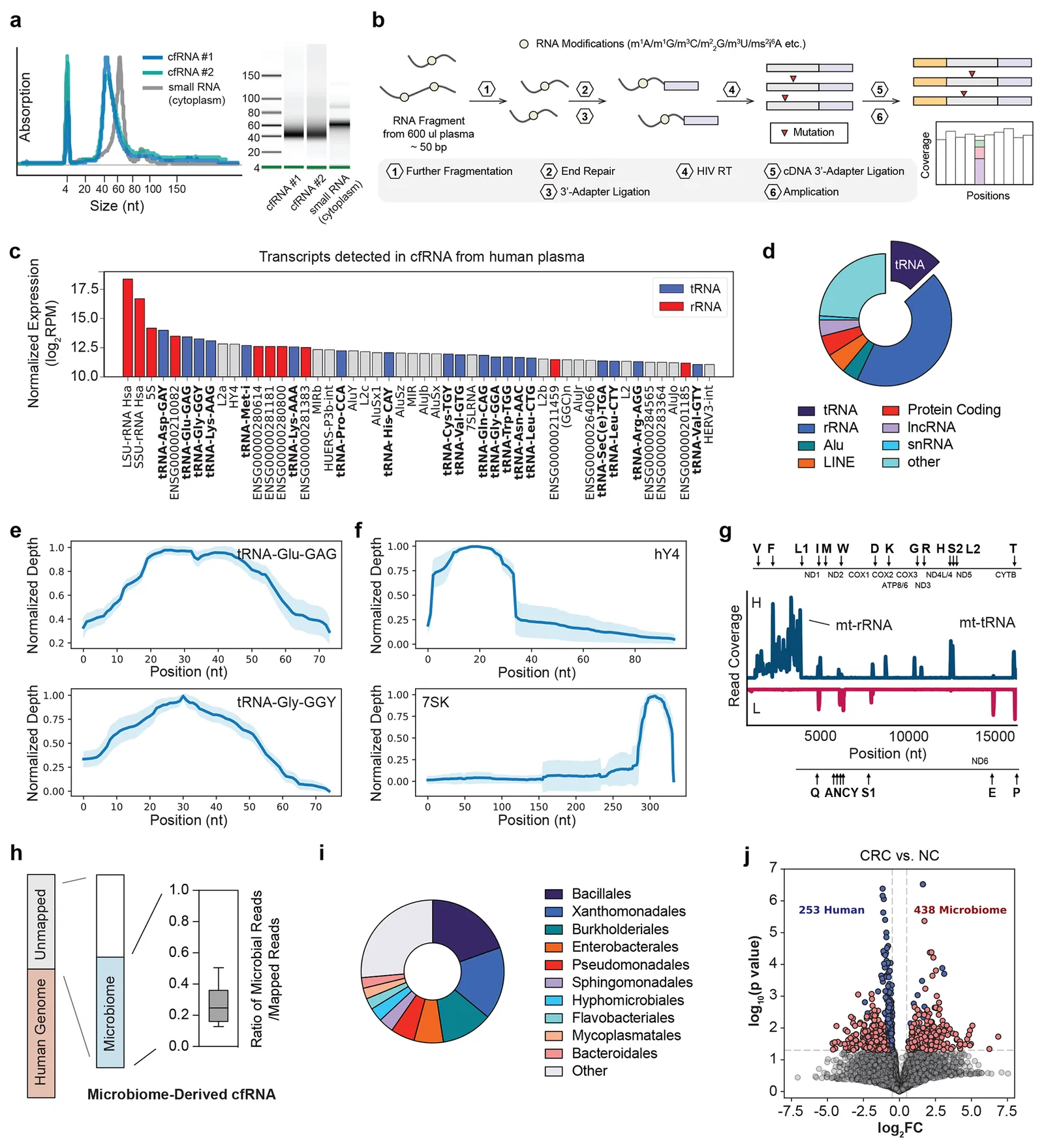
LIME-seq reveals the presence of host non-coding RNAs and microbiome-derived RNAs in human plasma cell-free RNA (cfRNA). **a**, High-resolution bioanalyzer assay showing input RNA sizes in LIME-seq libraries for human plasma cfRNA (from the blood of two non-cancer individuals) and HepG2 small RNA (< 200 nt, purified from total RNA), respectively. **b**, Schematic overview of LIME-seq. Light green circles denote diverse cfRNA modifications that induce mutation signatures with HIV RT. RNA/cDNA dual ligations capture mutation signals at reads end and internal positions. **c**, Bar plots showing relative expression levels of the top 50 abundant RNA transcripts in plasma cfRNA, indicating the dominance of tRNA and rRNA. **d**, Proportional distribution of all mapped reads classified by RNA species in plasma cfRNA LIME-seq libraries. **e**, Representative tRNA reads coverage obtained by LIME-seq in plasma cfRNA, showing intact coverage across host tRNA regions. **f**, Reads coverage for representative non-tRNA RNA species (e.g*.* 7SK and hY4 RNA) obtained by LIME-seq in plasma cfRNA, revealing incomplete coverage and indicating partial RNA degradation. For **e** and **f**, Y axis shows normalized reads coverage depth within each annotated RNA by RNA length (X axis). Data are presented as mean values +/− SD. **g**, LIME-seq reads coverage mapped to mitochondrial genome (plasma cfRNA), with blue and red denoting heavy and light mt-DNA strands. Accumulated reads coverage was observed in mt-rRNA and mt-tRNA regions. **h**, Left: stacked bar chart showing the proportional distribution of LIME-seq reads mapped to human genome versus unmapped reads aligned to microbial genomes (plasma cfRNA). Right: box plot of microbiome-related reads ratios within unmapped reads (*n* = 36 non-cancer individuals; box plots show median, 25th–75th percentiles, and whiskers for 10th–90th percentiles. **i**, Pie chart showing percentages of microbiome-derived cfRNA by the top 10 abundant microbial orders and others, based on LIME-seq. **j**, Volcano plot for differential expression of host and microbial cfRNA species assessed by LIME-seq, comparing CRC patients to non-cancer controls. Genes or genera with significant changes are highlighted (host: blue; microbiome: red). *p*-values were calculated using an unpaired t-test (two tailed)**.**

**Fig. 2 |:**
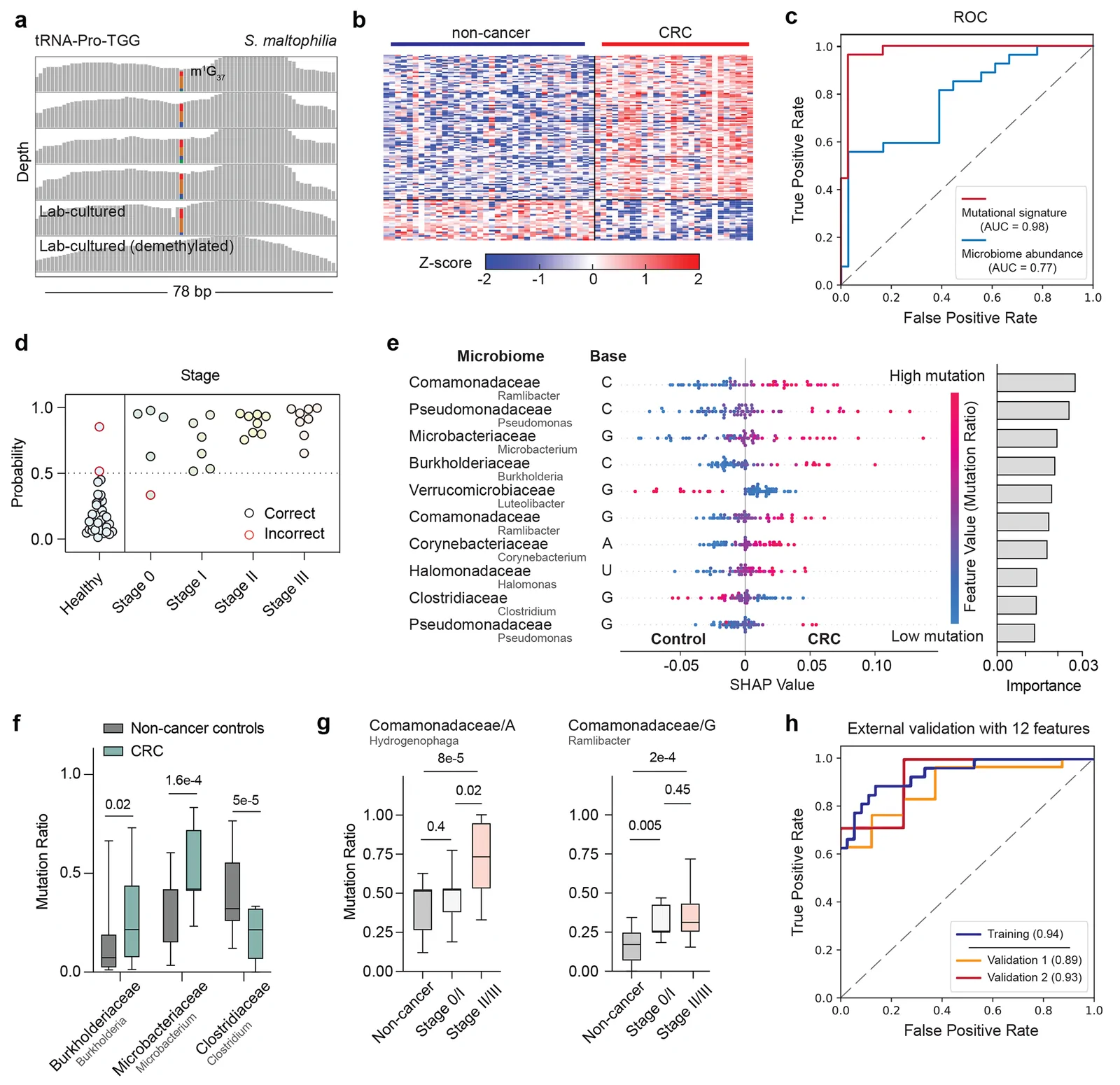
LIME-seq detects RNA modification signatures in microbiome-derived RNAs in plasma cfRNA, offering a set of biomarkers for CRC early diagnosis. **a**, IGV plot of a representative microbiome-derived tRNA region in *Stenotrophomonas maltophilia*, showing LIME-seq reads coverage and m^1^G_37_ mutation sites in plasma cfRNA and RNA isolated from the lab-cultured microbiome with and without demethylation. **b**, Heatmap showing differential mutation signatures in microbiome-derived RNAs of plasma cfRNA between CRC patients and non-cancer controls. **c**, Receiver Operating Characteristic curve (ROC) of the classifier showing how LIME-seq of plasma cfRNA distinguishes CRC patients from non-cancer controls, using microbiome-derived RNA abundance or mutation patterns, validated by LOOCV. **d**, Scatter plot showing high classifier sensitivity for predicting probabilities across different CRC stages, based on mutation patterns in microbiome-derived plasma cfRNA. **e**, SHAP values from the classifier model highlighting key mutation sites in 10 representative microbes that predominantly contribute to the CRC diagnostic model. **f**, Box plot comparing integral mutation patterns in microbiome-derived RNAs, showing significant differences between CRC patients and non-cancer individuals across the three representative microbes in plasma cfRNA, validating the CRC diagnostic model. *n* = 36 for non-cancer controls and *n* = 27 for CRC groups. **g**, Box plot showing stage-specific mutation pattern dynamics in microbiome-derived plasma cfRNA, using *Hydrogenophaga/A* and *Ramlibacter tataouinensis/G* as examples. Mutation pattern variations across different CRC stages indicate the potential for monitoring cancer-progression-related changes, *via* the CRC diagnostic model. *n* = 36 (control), *n* = 11 (stage 0/I), and *n* = 16 (stage II/III). Box plots showing the median, 25th–75th percentiles, and whiskers for 10th–90th percentiles. *p*-values were calculated using an unpaired t-test (two tailed)**. h,** ROC curves for the classifier based on 12 mutational signatures in microbiome-derived plasma cfRNA, detected by LIME-seq, for the detection of CRC patients in the discovery cohort, validation cohort 1, and validation cohort 2.

## Data Availability

The human genome reference (GRCh38.108) was used in this study. The raw and processed sequencing data have been deposited into the NCBI Gene Expression Omnibus (GEO) database with the accession number GSE264208.^64^
