# Supplementary material for: Modifications of microbiome-derived cell-free RNA in plasma discriminates colorectal cancer samples
Source: Nat Biotechnol. Author manuscript; Available in PMC 2026 Sep 29. (PMC13621429; doi:10.1038/s41587-025-02731-8)
Supplement: Supplementary file 1 [file NIHMS2210148-supplement-Supplemantary_Data_Fig.pdf]

Supplementary Data Fig. 1

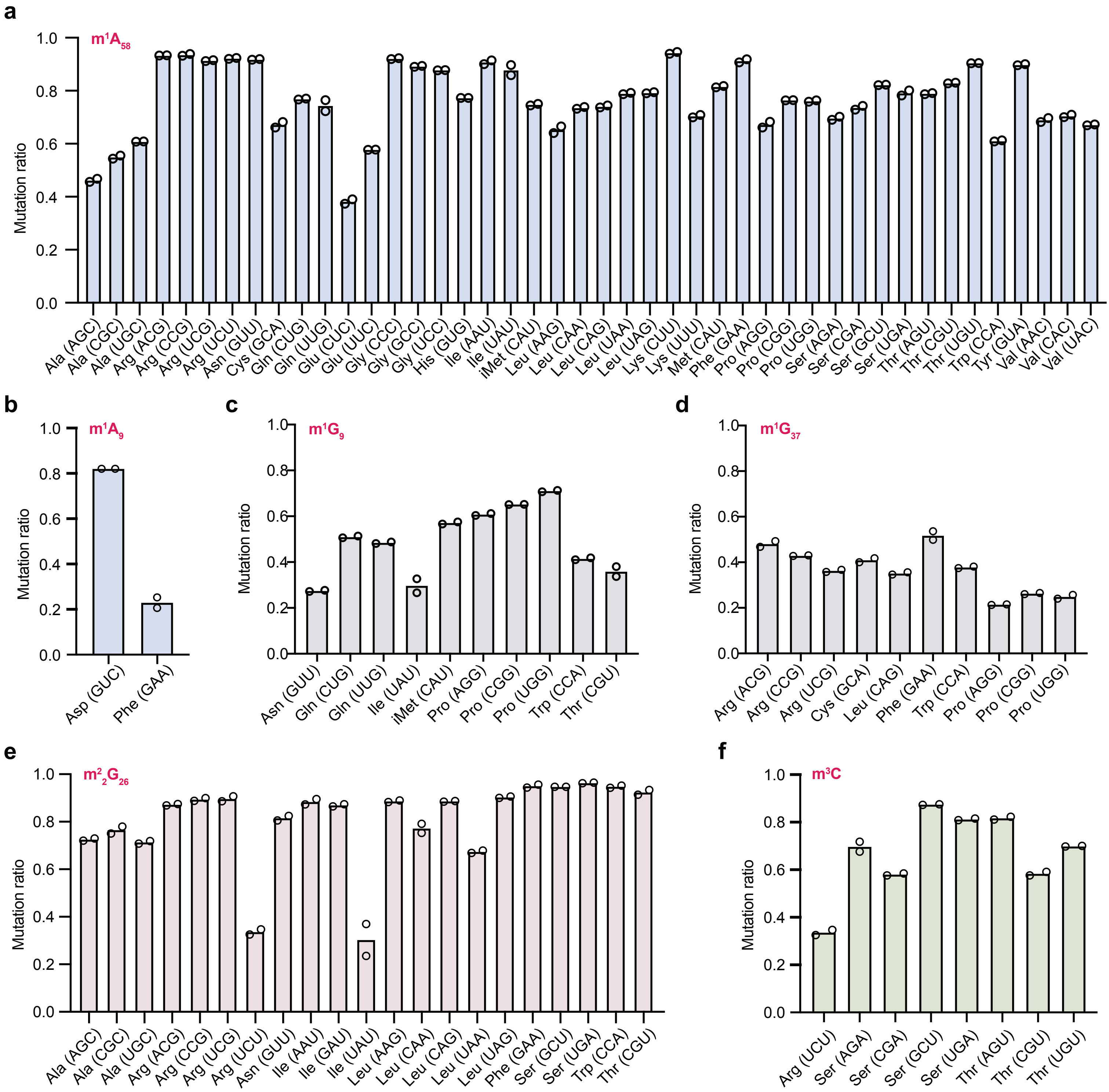

Supplementary Data Fig. 2

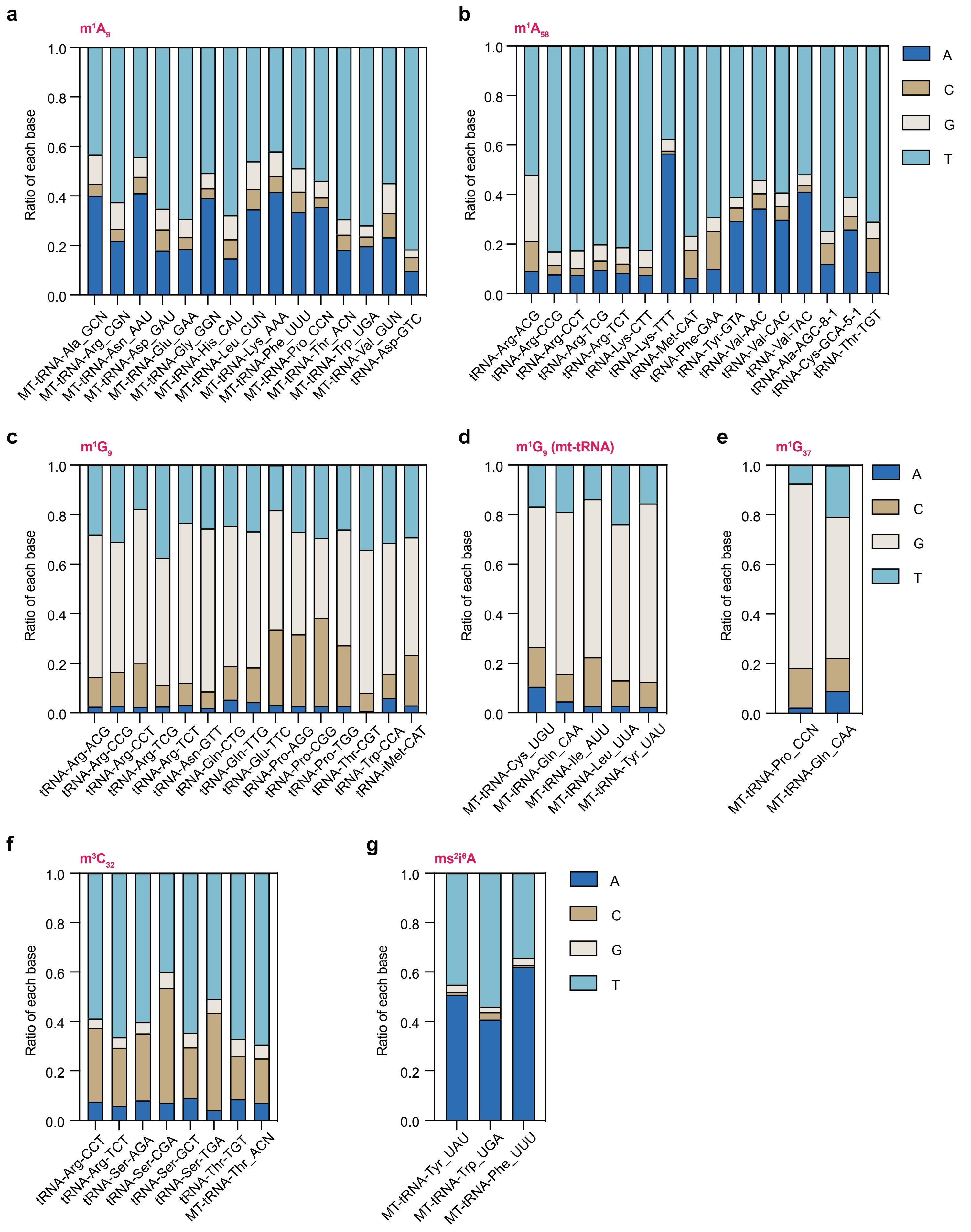

Supplementary Data Fig. 3

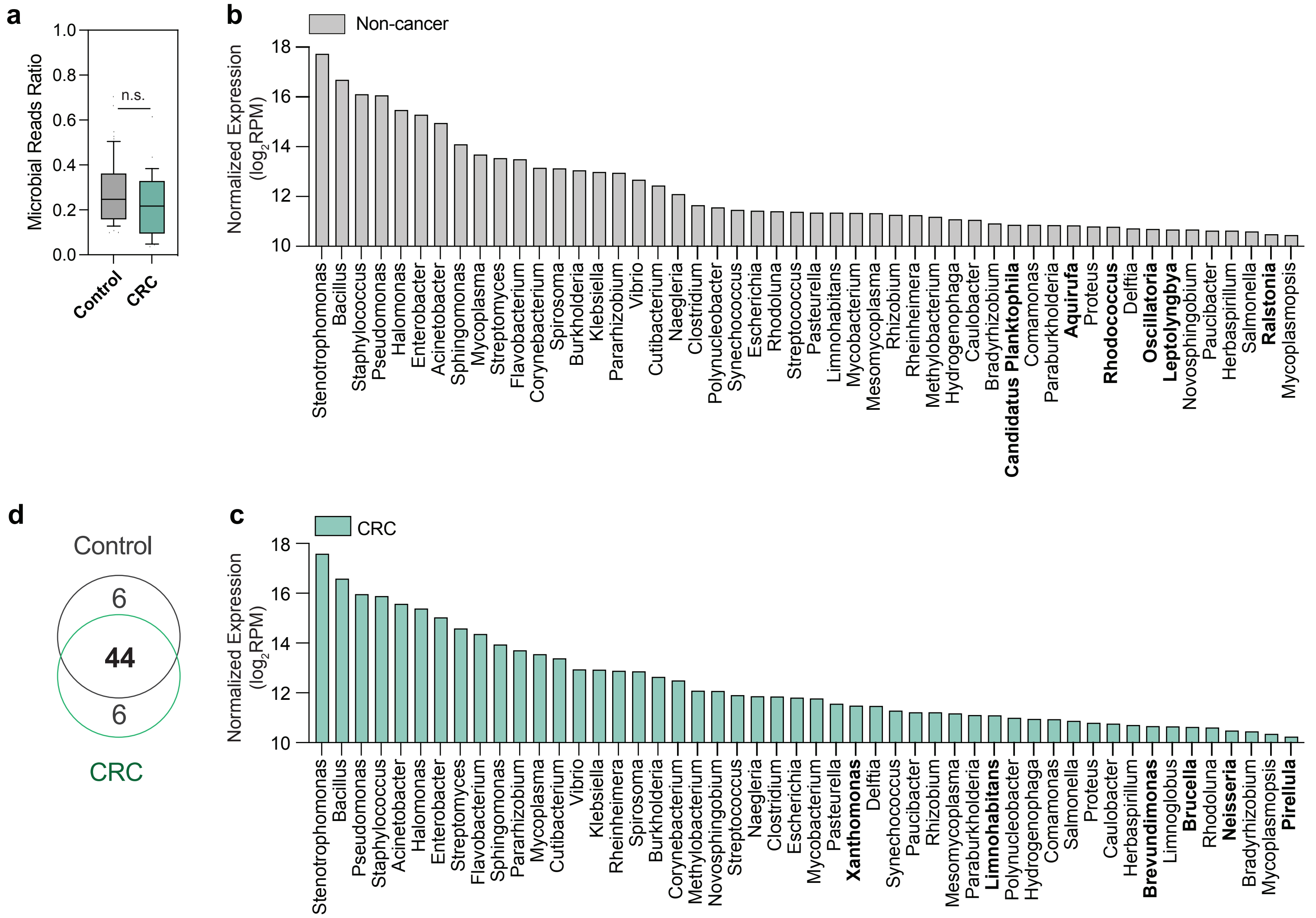

Supplementary Data Fig. 1 | Quantitation profile of RNA methylations in small RNAs from HepG2 cells by LIME-seq.

a–**f**, Barplot shows the mutation ratios of RNA methylation sites m^1A_{58} (**a**), m^1A_9 (**b**), m^1G_9 (**c**), m^1G_{37} (**d**), $m_2^2G_{26}$ (**e**), and m^3C (**f**) detected by LIME-seq in small RNAs from HEPG2 cells. Each data point represents one biological replicate.

Supplementary Data Fig. 2 | The methylation profile of host tRNAs in human plasma cell-free RNAs, revealed by LIME-seq.

a–**g**, Stacked bars show the mutation ratios of tRNA methylation sites m^1A_9 (**a**), m^1A_{58} (**b**), m^1G_9 (**c**), m^1G_9 (mitochondrial tRNA) (**d**), m^1G_{37} (**e**), m^3C_{32} (**f**) and ms^2i^6A (**g**) detected by LIME-seq in cfRNAs. For **a–g,** The ratios for different nucleobases are averaged from 36 non-cancer individuals.

Supplementary Data Fig. 3 | Comparison of microbiome-derived cfRNA origins in CRC patients versus non-cancer controls.

a, Boxplot compares the ratio of microbial reads from LIME-seq between non-cancer controls and CRC patients. Box plots show the median and interquartile range (25th to 75th percentile); whiskers extend from the 10th to 90th percentile, with n = 36 individuals for the control group and n = 27 individuals for the CRC group. *p*-values were calculated using an unpaired t-test (two tailed). **b**–**c**, Bar plot shows the relative abundance of the top 50 most abundant microbial species detected by LIME-seq in plasma samples from 36 non-cancer individuals (**b**) and 27 CRC patients (**c**). Abundance is determined by Log_2RPM values calculated using LIME-seq data and averaged from 35 non-cancer individuals and 27 cancer patients, respectively. **d**, Venn diagram shows the overlap of the top 50 most abundant microbial species in non-cancer individuals and patients with colorectal cancer (CRC).

Supplementary Table legends

Supplementary Table 1. Overview CRC patients and non-cancer controls participating in our study (training cohort). Mean values are accompanied by their 95% confidence intervals (CIs), indicated within brackets, to provide a statistical summary of the key measurements.

Supplementary Table 2. Top 100 significant sites in the microbiome for CRC detection as identified by the statistical model. These sites are categorized at the genus level, with references provided accordingly. *p*-values for differential methylation were calculated using a two-tailed Student's t-test.

Supplementary Table 3. Twenty-six significant microbiome genera identified by the statistical model. For references and detailed information, please refer to Supplementary Table 2**.**

Supplementary Table 4. Overview CRC patients and non-cancer controls participating in the validation cohort 1. Mean values are accompanied by their 95% confidence intervals (CIs), indicated within brackets, to provide a statistical summary of the key measurements.

Supplementary Table 5. Overview CRC patients and non-cancer controls participating in the validation cohort 2. Mean values are accompanied by their 95% confidence intervals (CIs), indicated within brackets, to provide a statistical summary of the key measurements.

Supplementary Table 6. Differential mutational sites in the microbiome between PDAC patient and controls . These sites are categorized at the genus level, with references provided accordingly. *p*-values for differential methylation were calculated using a two-tailed Student's t-test.
